# Inhibition of *Ehrlichia chaffeensis* infection by cell-permeable macrocyclic peptides that bind type IV secretion effector Etf-1

**DOI:** 10.1093/pnasnexus/pgad017

**Published:** 2023-01-27

**Authors:** Mingqun Lin, Amritendu Koley, Wenqing Zhang, Dehua Pei, Yasuko Rikihisa

**Affiliations:** Department of Veterinary Biosciences, The Ohio State University, 1925 Coffey Rd, Columbus, OH 43210, USA; Department of Chemistry and Biochemistry, The Ohio State University, 484 West 12th Avenue, Columbus, OH 43210, USA; Department of Veterinary Biosciences, The Ohio State University, 1925 Coffey Rd, Columbus, OH 43210, USA; Department of Chemistry and Biochemistry, The Ohio State University, 484 West 12th Avenue, Columbus, OH 43210, USA; Department of Veterinary Biosciences, The Ohio State University, 1925 Coffey Rd, Columbus, OH 43210, USA

**Keywords:** *Ehrlichia chaffeensis*, type IV secretion system, Etf-1, macrocyclic peptides, autophagy, Beclin 1

## Abstract

*Ehrlichia chaffeensis* is an obligatory intracellular bacterium that infects monocytes and macrophages, and causes human monocytic ehrlichiosis, an emerging life-threatening infectious disease. *Ehrlichia* translocated factor-1 (Etf-1), a type IV secretion system effector, is essential for *Ehrlichia* infection of host cells. Etf-1 translocates to mitochondria to block host apoptosis; furthermore, it can bind Beclin 1 (ATG6) to induce cellular autophagy and localize to *E. chaffeensis*-inclusion membrane to obtain host-cell cytoplasmic nutrients. In this study, we screened a synthetic library of over 320,000 cell-permeable macrocyclic peptides, which consist of an ensemble of random peptide sequences in the first ring and a small family of cell-penetrating peptides in the second ring, for Etf-1 binding. Library screening followed by hit optimization identified multiple Etf-1-binding peptides (with *K*_D_ values of 1–10 μM) that efficiently enter the cytosol of mammalian cells. Peptides B7, C8, B7-131-5, B7-133-3, and B7-133-8 significantly inhibited *Ehrlichia* infection of THP-1 cells. Mechanistic studies revealed that peptide B7 and its derivatives inhibited the binding of Etf-1 to Beclin 1, and Etf-1 localization to *E. chaffeensis*-inclusion membranes, but not Etf-1 localization to the mitochondria. Our results not only affirm the critical role of Etf-1 functions in *E. chaffeensis* infection, but also demonstrate the feasibility of developing macrocyclic peptides as powerful chemical probes and potential treatment of diseases caused by *Ehrlichia* and other intracellular pathogens.

**Significance Statement**

Infections by intracellular pathogens are difficult to treat because a drug molecule often needs to travel across multiple cell membranes to reach the disease-relevant target. The obligatory intracellular bacterium *Ehrlichia chaffeensis*, which causes human monocytic ehrlichiosis (HME), uses the type IV secretion system to deliver effector proteins like *Ehrlichia* translocated factor-1 (Etf-1) to establish intracellular infection. The present study demonstrates the development of cell-permeable macrocyclic peptides that bind to Etf-1 with high affinity and specificity. Several peptides blocked Etf-1 from binding to its host target protein Beclin 1 involved in cellular autophagy, thereby inhibited *E. chaffeensis* infection of host cells, suggesting that macrocyclic peptides can be developed as a therapeutic intervention for HME or other intracellular bacterial infections.

## Introduction

Human monocytic ehrlichiosis (HME) is one of the most prevalent, life-threatening, and emerging tick-borne diseases in the United States. HME is characterized by severe flu-like illness accompanied by hematologic abnormalities and signs of hepatitis (1–3). The presence of underlying illness or immunosuppression, elderly, and/or co-infection with other pathogens can exacerbate clinical signs of HME (4). Currently, the only effective therapy for HME is the broad-spectrum antibiotic doxycycline, which should be initiated early because delayed or lack of treatment can lead to severe complications or death. However, doxycycline is contraindicated for pregnant women and children, or those with drug allergies. Currently, no vaccine exists for HME, underscoring the importance of developing new therapeutics and preventive measures as tick-borne diseases have risen dramatically in the past 20 years and continued to rise (5, 6).

HME is caused by infection with *Ehrlichia chaffeensis*, an obligatory intracellular bacterium in the order Rickettsiales (7, 8). *E. chaffeensis* replicates within human monocytes/macrophages and forms numerous microcolonies called “morulae” or “inclusions” in specialized membrane-bound compartments. Inclusions provide a sanctuary for *E. chaffeensis* to avoid host innate-immune microbicidal mechanisms and acquire host cell–derived nutrients (9–11), which have early endosome-like characteristics, including the small GTPase RAB5 and RAB5 effectors EEA1 (early endosome antigen 1), VPS34 (the catalytic subunit of class III PtdIns3K), and vacuolar-type H^+^ ATPase, but lack late endosomal or lysosomal markers and NADPH oxidase components (12–15). In order to dysregulate or hijack host-cell functions for intracellular survival and replication, *E. chaffeensis* delivers Ehrlichial translocated factors (Etfs) into the host cell cytoplasm by a type IV secretion system (T4SS) (11).

Our studies indicate that Etf-1 plays a critical role for *E. chaffeensis* to establish intracellular infection: (i) ectopic overexpression of Etf-1 protein enhances *E. chaffeensis* infection (15); (ii) delivering anti-Etf-1 IgG into the cytoplasm of host cells inhibits *E. chaffeensis* infection (16); (iii) electroporation of *E. chaffeensis* with Etf-1-specific peptide nucleic acid that reduces Etf-1 mRNA and protein, significantly inhibits *E. chaffeensis* infection and ectopic expression Etf-1 complements this inhibition (17); and (iv) cytoplasmic delivery of anti-Etf-1 nanobodies by conjugating to a cyclic cell-penetrating peptide (CPP), inhibits *E. chaffeensis* infection in cell culture and in a mouse model (18). These data demonstrate Etf-1 as a valid target for therapeutic intervention against HME. However, the therapeutic application of anti-Etf-1 nanobody-CPP conjugates still faces significant challenges, as the large-scale production of the anti-Etf-1 nanobody is expensive and site-specific conjugation of a chemically synthesized CPP to a nanobody remains a major technical hurdle. As such, alternative modalities are needed for combating infection by obligatory intracellular pathogens.

Over the last two decades, macrocyclic peptides have emerged as a new class of drug modalities for targeting challenging protein targets such as those involved in protein–protein interactions (PPIs), because of their capability of binding to flat protein surfaces with high affinity and specificity (19–21). A key limitation of macrocyclic peptides is that they are generally impermeable to the cell membrane. To overcome this limitation, we previously designed cell-permeable macrocyclic peptides in which one ring contained a CPP for cellular entry while the other ring had a target-binding sequence (22). Further, we chemically synthesized large one bead–one compound (OBOC) libraries of cell-permeable macrocyclic peptides, by incorporating a small family of CPP motifs in one ring and a degenerate/random peptide sequence in the second ring. Screening of the OBOC libraries for binding against a protein target of interest identified cell-permeable and biologically active macrocyclic peptides (23–25). In some instances, the CPP motif also interacts with the target protein, making significant contributions to the overall binding affinity and specificity, in addition to ensuring cellular entry. In the current study, we sought to apply the OBOC library approach to identify cell-permeable macrocyclic peptides that block Etf-1 functions and *E. chaffeensis* infection.

## Results

### Identification of Etf-1-binding peptides by library screening

**Fig. 1.**
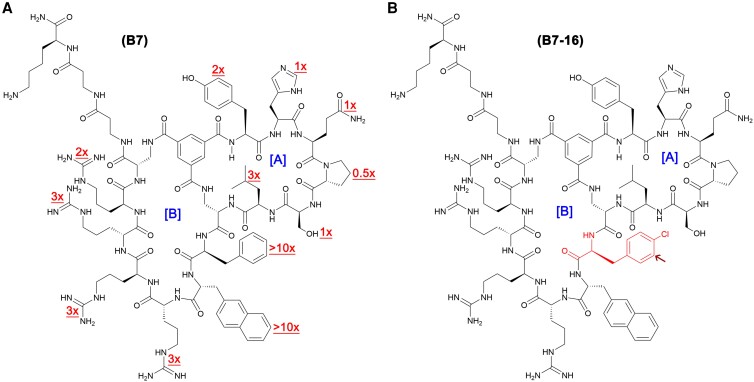
Structure of Etf-1-binding macrocyclic peptides B7 and B7-16. (A) Structure of peptide B7. A-ring is responsible for Etf-1 target engagement, whereas B-ring contains CPP sequences responsible for cell permeability. The value next to each residue (indicated by underlined texts in red fonts) of B7 represents the fold of reduction in Etf-1-binding affinity upon substitution of alanine (L- or D-) for that residue. (B) Structures of B7-16. The L-4-chlorophenylalanine residue in B7-16 is indicated by an arrow and highlighted in red.

To identify potential binders of Etf-1 protein, we first screened a previously reported cell-permeable macrocyclic peptide library (25), where one ring (A-ring) contained random peptide sequences of 3–6 residues for target engagement, while the other ring (B-ring) featured 12 different CPP sequences. Approximately, 320,000 beads were screened for binding to Etf-1 in three different rounds as described in the experimental section (25, 26), resulting in 43 hits. Sequence determination of the hits by mass spectrometry gave 30 complete sequences with high confidence (27) (Tables 1 and S1), and the structure of a hit peptide B7 was shown in Fig. 1A. Although the selected sequences do not show any obvious consensus, they exhibit some interesting trends. First, the selected sequences contain a much greater number of CPPs (26/30) that begin with hydrophobic residues (Phe, D-Phe, Nal, or D-Nal) than those that begin with Arg or D-Arg residues (4/30), even though the two types of CPPs were roughly equally populated in the original peptide library. This suggests that the CPP sequences in the B ring likely make significant contributions to Etf-1 binding. Second, the Etf-1-binding sequences in the A ring contain substantially more acidic residues (16 Asp and D-Glu residues) than basic residues [6L-ornithine (Orn) and D-Lys], despite an equal population of acidic and basic residues in the peptide library.

**Table 1.** Sequences and Etf-1-binding affinities of macrocyclic peptides used in this study^a^

Peptide ID	Sequences^b^	*K*_D_ (µM)^c^
B7	TMA-Tyr-His-Gln-pro-Ser-leu-Dap-Phe-nal-arg-Arg-arg-Arg-Dap	6.5 ± 0.7
C8	TMA-glu-val-Tyr-Trp-Gly-Dap-Phe-nal-arg-Arg-arg-Arg-Dap	5.0 ± 1.2
C17	TMA-glu-Ser-ala-phe-His-Ser-Dap-phe-Nal-Arg-arg-Arg-arg-Dap	>25
B7-16	TMA-Tyr-His-Gln-pro-Ser-leu-Dap-4-ClPhe-nal-arg-Arg-arg-Arg-Dap	2.0 ± 0.2
B7-95-1	TMA-Tyr-Asp-Orn-isoAsp-glu-Fpa-leu-Dap-4-ClPhe-nal-arg-Arg-arg-Arg-Dap	3.2
B7-95-2	TMA-Tyr-val-Asp-Asp-Orn-Fpa-leu-Dap-4-ClPhe-nal-arg-Arg-arg-Arg-Dap	0.74 ± 0.06
B7-131-1	TMA-Tyr-asn-nal-Ser-Phg-leu-leu-Dap-4-ClPhe-nal-arg-Arg-arg-Arg-Dap	1.7 ± 0.2
B7-131-4	TMA-Tyr-Fpa-Asp-Phg-tyr-isoAsp-leu-Dap-4-ClPhe-nal-arg-Arg-arg-Arg-Dap	∼1.5
B7-131-5	TMA-Tyr-His-pro-nal-Asp-Ala-leu-Dap-4-ClPhe-nal-arg-Arg-arg-Arg-Dap	2.9 ± 0.4
B7-133-3	TMA-Tyr-phe-Asp-Nle-asn-leu-Dap-4-ClPhe-nal-arg-Arg-arg-Arg-Dap	2.4 ± 0.3
B7-133-8	TMA-Tyr-nal-Gln-homoAla-pro-leu-Dap-4-ClPhe-nal-arg-Arg-arg-Arg-Dap	1.3 ± 0.1
B7-133-9	TMA-Tyr-val-leu-tyr-Fpa-leu-Dap-4-ClPhe-nal-arg-Arg-arg-Arg-Dap	2.2 ± 0.3

a Etf-1-binding macrocyclic peptides used in this study were obtained from the first-round library screening (B7–C17), through alanine scanning and optimization (B7-16), and from the second-generation library screening (B7-95-1 to B7-133-9). The complete lists of peptides identified were summarized in supplementary Tables S1–S3.

b Three-letter abbreviations with capitalized first letter are used for L-amino acids, while three-letter abbreviations in lowercase letters for D-amino acids. TMA, trimesic acid; Dap, 2,3-diaminopropionic acid; Fpa, L-4-fluorophenylalanine; Nal, L-2-naphthylalanine; nal, D-2-naphthylalanine; Nle, L-norleucine; Orn, L-ornithine; Phg, L-phenylglycine; 4-ClPhe, L-4-chlorophenylalanine; isoAsp, aspartic acid α-tert-butyl ester; Fpa, L-4-fluorophenylalanine; homoAla, D-β-homo-alanine.

c Etf-1-binding affinity was measured by the FP assay, using peptides labeled with FAM through a C-terminal β-Ala-β-Ala-Lys linker sequence.

**Fig. 2.**
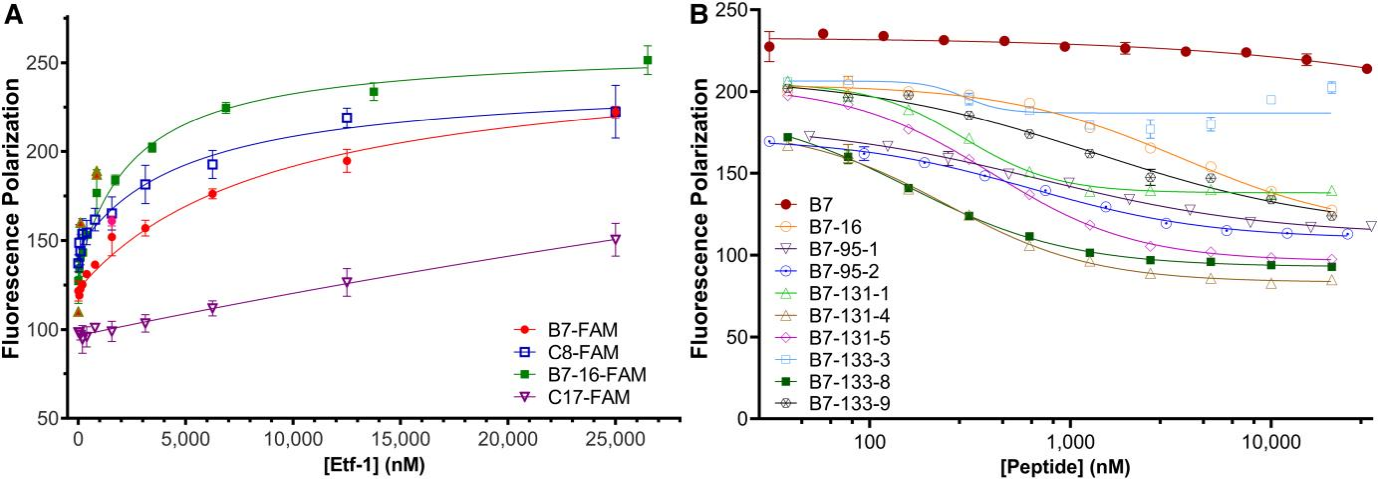
Determination of binding affinity of Etf-1-binding peptides by FP assay. (A) Binding of FAM-labeled B7, C8, C17, and B7-16 to Etf-1 as monitored by FP assay. FAM-labeled peptide (50 nM) was incubated with serially diluted Etf-1 (starting from 25–30 μM in PBS, pH 7.4, supplemented with 5 mM DTT and 0.01% Triton X-100) for 1 h at RT. FP values were measured on a Tecan Infinite M1000 Pro plate reader. Titration curves (*n* ≥ 3) were fitted using GraphPad Prism and analyzed with TraceDrawer to calculate dissociation constants (*K*_D_). (B) FP-based assay of competition for Etf-1-binding among second-generation peptides and B7. Etf-1 (500 nM) and FAM-labeled B7-133-8 (50 nM) were used in FP competition assay, and the IC_50_ values were determined by titrating the unlabeled competitor peptides (serially diluted concentration shown in nM in *X*-axis). Data were analyzed using GraphPad Prism and fitted to a four-parameter variable slope equation to determine IC_50_ values.

We selected nine of the peptides (B7, B13, B17, B22, C6, C8, C9, C17, and C19) for re-synthesis and further testing for Etf-1 binding, because these peptides have longer peptide sequences in the A ring (4–6 residues) and are expected to bind to Etf-1 with higher affinity. The peptides were labeled at their C-terminus with carboxyfluorescein (FAM) through a β-Ala-β-Ala-Lys linker and evaluated for binding to Etf-1 by using a fluorescence polarization (FP) assay. Among the nine peptides tested, peptides B7 and C8 showed relatively potent binding to Etf-1, with *K*_D_ values of 6.5 ± 0.7 and 5.0 ± 1.2 µM, respectively (Fig. 2A and Table 1). Note that peptides B7 and C8 contain the same CPP sequence, Phe-D-Nal-D-Arg-Arg-D-Arg-Arg, in the B ring. The other seven peptides showed only weak binding (*K*_D_ > 25 μM, Table S1) with no saturation reached at the highest Etf-1 concentration tested (25 μM), including peptide C17 (Fig. 2A) that was selected as a negative control in future assays. This outcome is not unexpected, as screening OBOC libraries is often complicated by false positives, due to the presence of high ligand densities on library beads (28).

### Initial optimization of peptide B7 for improvement of Etf-1-binding affinity

We chose peptide B7 for optimization and further studies because of its better overall biological activity than C8 in our preliminary cellular studies (see below). Since the three-dimensional structure of Etf-1 is not yet available, we used the “alanine scan” approach to determine the residues that are critical for Etf-1 binding, by replacing each amino acid of B7 with L- or D-alanine. The alanine-scan peptides (peptides B7-1 to B7-12, Table S2) were similarly labeled with FAM and their binding affinities for Etf-1 were determined by the FP assay. Surprisingly, replacement of the residues in the A ring by Ala (or D-Ala) had relatively minor effect on the Etf-1-binding affinity (Table S2, peptides B7-1 to B7-6). The greatest reduction in the binding affinity (three-fold) was when D-Leu was replaced with D-Ala followed by the mutation of Tyr or His into Ala (∼two-fold), while replacement of other residues in the A ring (Gln − D-Pro − Ser) had minimal effect on the binding affinity for Etf-1 (Table S2 and Fig. 1A). On the other hand, replacement of the B-ring residues had much greater effect on Etf-1 binding. Substitution of Ala (or D-Ala) for either Phe or D-Nal largely abolished Etf-1 binding (>10-fold reduction in Etf-1 binding), while removing each of the arginine side chains resulted in 2- to 5-fold loss in binding affinity (Table S2, peptides B7-7 to B7-12, and Fig. 1A). These results are consistent with the library screening data and indicate that the B ring contributes most of the Etf-1-binding energy, whereas some of the A ring residues (Gln − D-Pro − Ser) are not engaged in significant interactions with the Etf-1 surface.

Having identified the B-ring residues as more critical for Etf-1 binding, we attempted to optimize B7 for Etf-1 binding by replacing the two key hydrophobic residues (Phe and D-Nal) with commercially available structural analogs. Although the arginine residues are also important for Etf-1 binding, we opted to keep them unchanged because of their critical roles in cellular entry and the lack of commercially available arginine analogs. Thus, we first elongated or shortened the side chain of Phe by one carbon atom. Replacement of Phe with L-4-methylphenylalanine (4-MePhe) or L-phenylglycine (Phg) had no significant effect on the binding affinity (Table S2, peptides B7-13 and B7-14); however, substitution of L-homophenylalanine (HomoPhe) increased the Etf-1-binding affinity by 2-fold (peptide B7-15). We next replaced Phe with halogenated phenylalanine derivatives including L-4-chlorophenylalanine (4-ClPhe; peptide B7-16), L-3-chlorophenylalanine (3-ClPhe; peptide B7-17), L-4-fluorophenylalanine (Fpa; peptide B7-18), L-3,4-dichlorophenylalanine (Cl_2_Phe; peptide B7-19), or L-4-trifluoromethylphenylalanine (4-CF_3_Phe; peptide B7-20). We also replaced Phe with the larger, more hydrophobic L-2-Nal residue (peptide B7-21). Etf-1-binding affinities were evaluated by FP assay using C-terminal FAM-labeled peptides (Tables 1 and S2, with an example of B7-16 shown in Fig. 2A). Several of the resulting peptides showed three- to four-fold greater binding affinity than B7 (Fig. 1A). We chose peptide B7-16 (peptide structure shown in Fig. 1B) for further optimization because of its balance in potency (*K*_D_ = 2.0 ± 0.2 µM) and hydrophobicity, whereas some of the other peptides (B7-19 to B7-21) have more hydrophobic residues at this position (and potentially poorer aqueous solubility). Attempts to further improve the potency of B7-16 by substituting 3-(3-benzothienyl)-D-alanine (bta; peptide B7-22) or D-1-naphthylalanine (1-nal; peptide B7-23) for D-2-Nal were unsuccessful.

### Further optimization of peptide B7-16 by screening a second-generation library

To further improve the binding affinity and potentially the cell permeability of B7-16, we designed and synthesized a second-generation peptide library by replacing the His-Gln-D-Pro-Ser motif in the A ring of B7-16 with a randomized peptide sequence of three to five residues (Fig. S1). Each of the randomized positions was constructed with the following set of amino acid building blocks (total 29): 7 proteinogenic L-amino acids (Gly, Ala, Ser, Ile, Asp, Gln, and His), 12 D-amino acids (D-Ala, D-Pro, D-Val, D-Thr, D-Leu, D-Asn, D-Lys, D-Glu, D-Phe, D-Arg, D-Tyr, and D-Trp), and 10 noncanonical amino acids [D-β-homo-alanine (homoAla), L-homo-proline (Pip), cis-2-aminocyclopentylcarboxylic acid (cis-AcPc), aspartic acid α-tert-butyl ester (isoAsp), L-phenylglycine (Phg), D-2-naphthylalanine (D-Nal), L-4-fluorophenylalanine (4-Fpa), L-norleucine (Nle), and L-ornithine (Orn)]. The library was synthesized in the OBOC format on TentaGel S resin and has a theoretical diversity of 2.12 × 10^7^, although only ∼27% of the theoretical sequence space was covered by the amount of resin employed (2.0 g or 5.7 × 10^6^ beads).

The library was similarly screened for binding to Etf-1 but under more stringent conditions, by using 100 and 25 nM biotinylated Etf-1 for magnetic sorting and the streptavidin-alkaline phosphatase (SA-AP) assay, respectively. After these two rounds of screening, we obtained ∼170 positive beads, which were too many for individual synthesis and analysis. We, therefore, subjected the ∼170 beads to a third round of screening by the SA-AP assay, during which the reaction solution contained 100 nM peptide B7-16 as a competitor. It was anticipated that only the strongest binders would be able to outcompete B7-16 for binding to Etf-1, recruit SA-AP to the surfaces of these beads, and result in coloration of the beads, whereas beads containing weaker binders would stay colorless under these conditions. A potential caveat is that a weaker binder may still produce a positive bead if it binds to Etf-1 at a different site(s) from B7-16. Gratifyingly, the third round of screening reduced the number of positive beads from ∼170 to 27. For 17 out of the 27 beads, peptide sequencing by mass spectrometry was successful, resulting in unambiguous, complete peptide sequences (Table S3).

We individually synthesized 16 of the 17 peptides, labeled them with FAM at a C-terminus lysine residue, and tested them for Etf-1 binding by the FP assay. The synthesis of peptide B7-131-6 failed, thus this peptide was not included in future studies. Most of the peptides bound to Etf-1 with similar affinity to B7-16, showing *K*_D_ values 0.74–8.8 μM (Tables 1 and S3). One peptide (B7-133-5) bound with significantly lower affinity (*K*_D_ > 10 μM) than B7-16, while two of the peptides (B7-131-3 and B7-131-6) failed to produce meaningful binding curves (presumably due to solubility issues). To confirm Etf-1 binding and determine whether the above peptides bind to the same site as B7 and B7-16, we selected some of the more potent binders from above (B7-95-1, B7-95-2, B7-131-1, B7-131-3, B7-131-4, B7-131-5, B7-33-8, and B7-133-9) and performed an FP-based competition assay. Competition for Etf-1 binding was observed in all cases (peptide pairs) tested (Fig. 2B), suggesting that the B7 series of peptides bind to the same (or overlapping) site on Etf-1. Interestingly, most of the peptides showed IC_50_ values that were significantly lower than the corresponding *K*_D_ values derived from the FP assay (e.g. IC_50_ = 0.11 ± 0.02 μM vs *K*_D_ = 1.27 ± 0.08 μM for B7-133-8) (Table S3 and Fig. 2B).

### Etf-1-binding peptides enter host cell cytosol efficiently

**Fig. 3.**
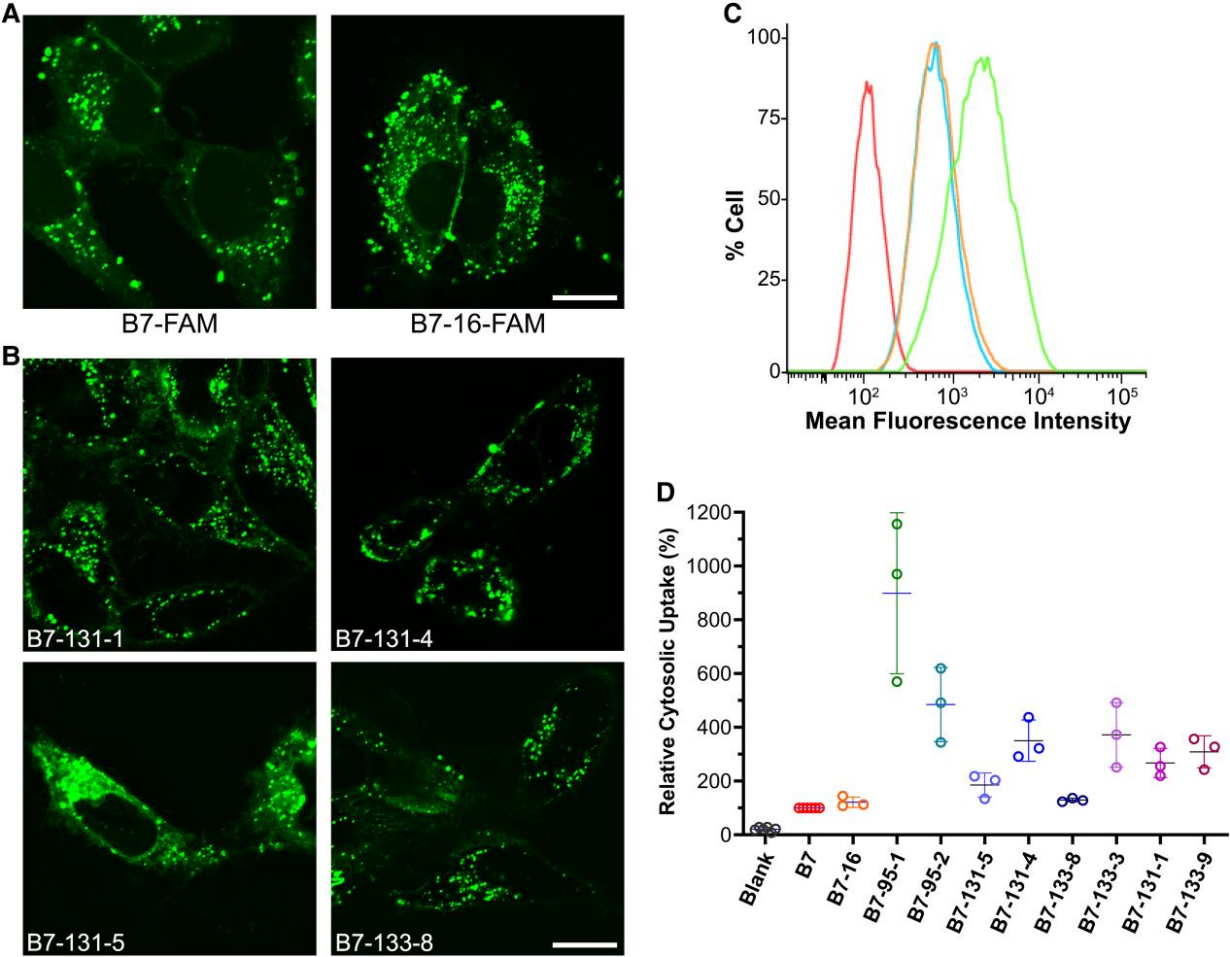
Cytosolic entry efficiency of Etf-1-binding peptides. (A and B) Representative live-cell confocal images of HeLa cells treated with FAM-labeled peptides. HeLa cells were treated for 2 h at 37°C with 5 µM B7-FAM and B7-16-FAM (A), or 2 μM FAM-labeled second-generation peptides (B). Scale bar, 5 µm. (C) Representative raw flow cytometry data of HeLa cells after treatment with 5 µM B7-NF (cyan), B7-16-NF (orange), CPP12-NF (green), or DMSO (red) for 2 h. (D) Relative MFI values of HeLa cells after treatment with 5 µM NF-labeled B7/B7-16 or 2 µM second-generation peptides for 2 h at 37°C in the presence of 10% FBS. All values are relative to that of B7 (100%).

The most potent peptides obtained from the first- and second-generation libraries, including B7, C8, B7-16, B7-95-1, B7-95-2, B7-131-1, B7-131-4, B7-131-5, B7-133-3, B7-133-8, B7-133-9, and negative control C17, were chosen for further cell-based evaluation. We first assessed the cellular entry efficiency of these peptides by live-cell confocal microscopy. HeLa (human cervical cancer) cells were incubated for 2 h at 37°C with FAM-labeled peptides. After treatment with 5 (B7 and B7-16) or 2 μM peptide (B7-131-1, B7-131-4, B7-131-5, B7-133-3, B7-133-8, and B7-133-9), almost all cells exhibited a combination of intense punctate fluorescence and diffuse but less intense fluorescence, especially in the cytoplasmic region (Fig. 3A and B). The punctate fluorescence likely reflects peptides still entrapped inside the endolysosomal pathway, while the diffuse fluorescence indicates that a fraction of the internalized peptides had reached the cytosol. To quantify their cytosolic entry, HeLa cells were incubated for 2 h with 5(6)-naphthofluorescein (NF)-labeled peptides and analyzed by flow cytometry. NF is a pH sensitive dye that fluoresces in the neutral environment of the cytosol and nucleus but not inside the acidic environment of the endosome or lysosome. As such, the mean fluorescence intensity (MFI) value of the treated cells reflects the concentration of peptides that have successfully reached the cytosol. A highly efficient cyclic CPP, CPP12 (29, 30), was used as a positive control. Cells treated with 5 µM B7-NF or B7-16-NF showed MFI values at 50–60% of CPP12 (Fig. 3C). Fortuitously, all peptides derived from the second-generation library had improved cytosolic entry efficiency as well, showing MFI values that were 1.3- to 9-fold higher than that of B7 (Fig. 3D). Despite higher cytosolic uptake efficiencies, preliminary results using B7-95-1 and B7-95-2 did not have improved inhibitory effects on *E. chaffeensis* infection over B7 or B7-16 (see Fig. S3); therefore, they are not included in further microscopic and cytotoxicity studies.

### Etf-1-binding peptides have varying cytotoxicity and are proteolytically stable

**Fig. 4.**
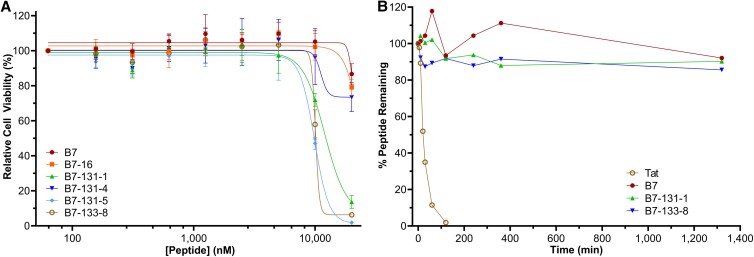
Cytotoxicity and proteolytic stability of Etf-1-binding peptides. (A) Effect of Etf-1-binding peptides on the viability of THP-1 cells as determined by CellTiter-Glo luminescent assay. Relative luminescence was plotted as function of the peptide concentration, with the cell viability of DMSO control group set as 100%. (B) Plot of the amount of intact peptide remaining as a function of the incubation time in 25% human serum. All values are normalized to that of peptide at time 0 (100%).

THP-1 (human promyelocytic leukemia) cells are widely used as *E. chaffeensis* infection studies of human monocytes in vitro, since initial characterization of infection (31). We tested peptides B7, B7-16, B7-131-1, B7-131-4, B7-131-5, and B7-133-8 for potential cytotoxicity against THP-1 and HeLa cells by using the CellTiter-Glo 2.0 assay. Peptides B7, B7-16, and B7-131-4 showed no cytotoxicity up to 10 μM and slightly reduced the cell viability at 20 μM after the 2-day treatment period (Fig. 4A). Unfortunately, the second-generation peptides (e.g. B7-131-1, B7-131-5, and B7-133-8) are generally more cytotoxic than B7 and B7-16, resulting in greater loss of cell viability at 20 µM (Fig. 4A). These peptides also caused significant release of lactate dehydrogenase (LDH; Fig. S2), suggesting that they cause plasma membrane damage, presumably because of greater hydrophobicity. Therefore, for all subsequent cell-based infection assays, the highest peptide concentration employed was 10 μM.

The serum stability of three representative peptides (B7, B7-131-1, and B7-133-8) was assessed by incubating the peptides in human serum at 37°C and monitoring the amount of intact peptide at various time points by ultra-performance liquid chromatography (UPLC). All three peptides were found to be proteolytically stable under this condition, showing <20% degradation after 22 h (Fig. 4B). In comparison, the linear peptide Tat (cell-permeable peptide of HIV) was completely degraded within the first 120 min of incubation (Fig. 4B).

### Inhibition of *E. chaffeensis* infection by Etf-1-binding peptides

Our previous studies demonstrated that successful *E. chaffeensis* infection of the host cells depends on *E. chaffeensis* T4SS effector Etf-1 (15, 16). Therefore, we investigated whether these Etf-1-binding peptides can prevent the proliferation of *E. chaffeensis* inside host cells. THP-1 cells were infected with *E. chaffeensis*, then treated with the peptides at 30 min post infection (pi) for 2 days. Intracellular infection and proliferation of *E. chaffeensis* inside host THP-1 cells were assessed using quantitative PCR (qPCR) analysis based on *E. chaffeensis* 16S rRNA gene normalized by human actin (*ACT*) gene.

**Fig. 5.**
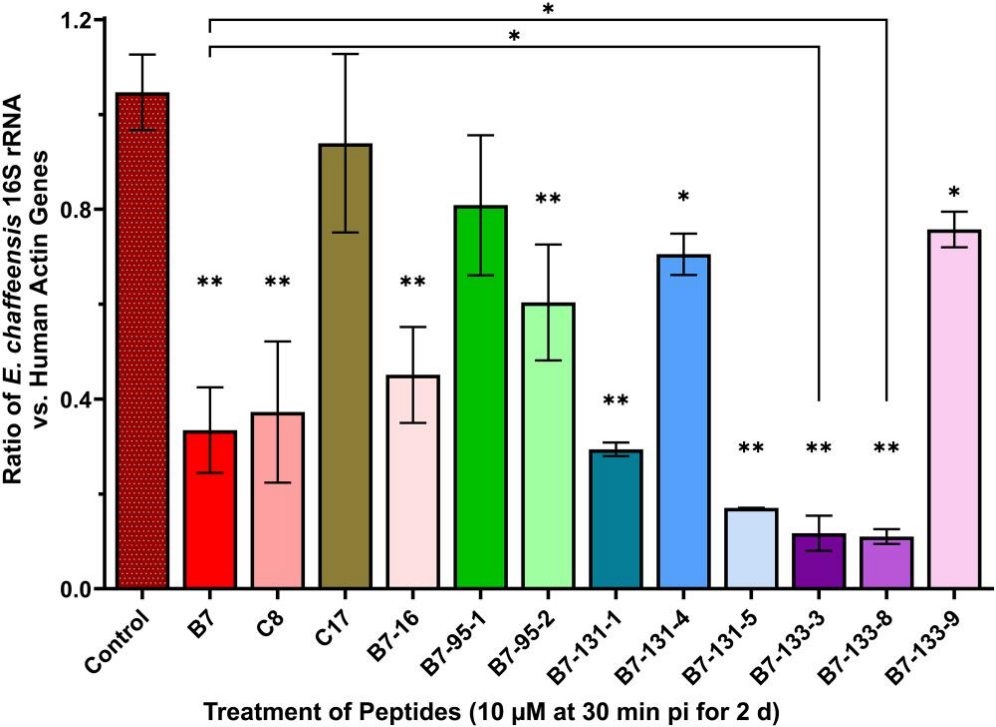
Effects of Etf-1-binding peptides on *E. chaffeensis* infection of THP-1 cells. *Ehrlichia chaffeensis*-infected THP-1 cells at 3 h pi were incubated with 10 μM of Etf-1-binding macrocyclic peptide or DMSO control for 2 days. DNA was extracted from each sample using Qiagen blood mini kit, and subjected to qPCR analysis using *E. chaffeensis* 16S rRNA normalized to human actin genes. **P < 0.01; *P < 0.05; one-way ANOVA compared with the control group or B7 treatment group (indicated by line brackets).

Compared with DMSO solvent-treated control group, at 1 μM, none of these Etf-1-binding peptides inhibited *E. chaffeensis* infection of THP-1 cells (Fig. S3). However, at 10 μM, most Etf-1-binding peptides, including B7, C8, B7-16, B7-95-2, B7-131-1, B7-131-5, B7-133-3, and B7-133-8, significantly inhibited *E. chaffeensis* infection in THP-1 cells (Fig. 5). Control peptide C17 had no effect on *E. chaffeensis* infection. Interestingly, although B7-16 and several peptides from the 2nd generation library have improved in vitro Etf-1-binding affinity and/or cell permeability over B7 (Table 1, Fig. 2), only peptides B7-133-3 and B7-133-8 showed significantly more potent inhibition of *E. chaffeensis* proliferation than peptide B7 (Fig. 5). Peptides B7, B7-133-3, and B7-133-8 inhibited *E. chaffeensis* infection in a dose-dependent manner over the concentration range (1, 5, and 10 μM) tested (Fig. S3).

Morphological examination of *E. chaffeensis* infection of THP-1 cells by Diff-Quik staining corroborated with the inhibitory and cytotoxic effects of these peptides detected by qPCR analysis. In control groups or peptides that did not inhibit *E. chaffeensis* infection like C17 or B7-95-1, *E. chaffeensis* organisms can be identified as generally round but sometimes pleomorphic cocci, which are stained dark blue to purple, and enclosed in spherical membrane-bound vacuoles called morulae in the cytoplasm (Fig. S4) (32, 33). Etf-1-binding peptides, including B7, B7-16, C8, B7-95-2, B7-131-1, 3-151-8, 1-133-3, and B7-133-8, significantly inhibited *E. chaffeensis* infection in THP-1 cells, as shown by the reduction of numbers and sizes of *E. chaffeensis* morulae (Fig. S4). Peptides B7-131-1, 3-151-5, and B7-133-8 showed certain degrees of cytotoxicity, since more cell lysis were observed in the treated groups as demonstrated in viability and LDH release assays (Figs. 4 and S2). However, the other three second-generation peptides, B7-95-1, B7-131-4, and B7-133-9, despite of higher binding affinity and cellular permeability, only slightly reduced *E. chaffeensis* infection (Figs. 5, S3, and S4). In conclusion, several second-generation Etf-1-binding peptides achieved greater inhibition on *E. chaffeensis* infection. However, peptide B7 had potent inhibition on bacterial proliferation with the least cytotoxicity on host cells; and therefore, was selected for following studies to characterize its inhibitory mechanisms.

### B7 colocalizes with Etf-1 at host cell mitochondria and *E. chaffeensis*-containing inclusions

In *E. chaffeensis*-infected cells, Etf-1 is secreted into the host cytoplasm, and localizes at two distinct intracellular sites to carry out two distinct activities, therefore regulating host cells to facilitate *E. chaffeensis* infection. First, Etf-1 localizes to mitochondria, where it upregulates Mn-superoxide dismutase to reduce reactive oxygen species level, thereby blocks host-cell apoptosis, allowing intracellular proliferation of *E. chaffeensis* (Fig. S5) (16). Second, Etf-1 interacts with RAB5-PIK3C3 complex, including RAB5, BECN1, and VPS34 (subunit of class III PtdIns3K, PI3KC3) to induce RAB5-regulated autophagosome formation, and localizes to the ATG5-positive pre-autophagosomes (15, 34). Etf-1-positive autophagosomes can fuse with *E. chaffeensis*-containing inclusions to deliver host cytosolic catabolites as nutrients into bacterial inclusions, therefore, a part of Etf-1 localizes on the inclusion membranes (Fig. S5) (15, 34).

**Fig. 6.**
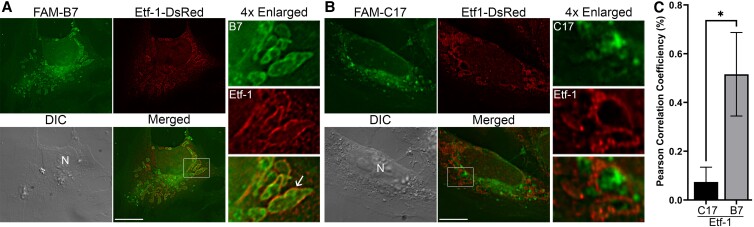
Colocalization of peptides with Etf1-DsRed at mitochondria in transfected RF/6A cells. RF/6A cells were seeded onto a coverslip in a 12-well plate for 1 day, and transfected with Etf-1-DsRed by Fugene HD reagent. At 2 days pt, cells were incubated with 2 μM FAM-labeled B7 or C17 for 2 h. Cells were fixed and observed under DeltaVision microscope. (A and B) The boxed area in the merged images is enlarged 4× at right. Arrow indicates the colocalization of Etf-1 with FAM-B7. Images are representative of three independent experiments. N, nucleus; Bar, 10 μm. (C) Colocalization of FAM-B7 or FAM-C17 with Etf-1 protein was analyzed with Pearson's correlation coefficient using SoftWoRx software from 10 transfected RF/6A cells per group from three independent experiments. *P < 0.01 by Student's unpaired t test.

We first used monkey endothelial RF/6A cells for unambiguous localization analysis by immunofluorescence labeling, since they are thinly spread adherent cells, can be infected with *E. chaffeensis* at high levels, and easily transfected (35). To confirm the binding of these peptides to Etf-1 within the host cells, Etf-1-DsRed fusion protein was ectopically expressed in RF/6A cells for 2 d, then incubated with FAM-labeled peptides for 2 h. Under this treatment condition, FAM-labeled peptides are internalized into the host cells and released into cytoplasm (Fig. 3), which can bind to Etf-1 proteins but are not expected to alter the protein localization. Immunofluorescence microscopy showed that, ectopically expressed Etf-1 protein was translocated to mitochondria, which appeared as filamentous structures in RF/6A cells (Fig. 6A and B). FAM-B7 was internalized into cytosol and mostly colocalized with Etf-1-DsRed at mitochondria with distinctive filamentous labeling (∼52% as measured by Pearson's correlation coefficiency; Fig. 6A and C). The control peptide FAM-C17, which does not bind Etf-1, did not colocalize with the Etf-1 and remained diffused in cytosol (Fig. 6B and C).

To examine whether peptide B7 binds to native Etf-1 protein in *E. chaffeensis*-infected cells, infected RF/6A or THP-1 cells at 2 d pi were incubated with FAM-labeled B7 peptide for 2 h. Fluorescence microscopy showed FAM-B7 was taken up by nearly 100% of THP-1 or RF/6A cells, and localized within cytosolic regions in uninfected cells (Fig. S6). In *E. chaffeensis*-infected cells, FAM-B7 was localized to distinct filamentous-shaped mitochondria in RF/6A cells (Fig. S6A, open arrows), as well as membranes surrounding *E. chaffeensis* inclusions in both RF/6A and THP-1 cells (Fig. S6, thin arrows). The distributions of FAM-B7 in *E. chaffeensis*-infected cells were similar to those of Etf-1 protein (Fig. S5), suggesting that FAM-B7 can bind to native Etf-1 protein translocated to both mitochondria and autophagosomes that traffic to *E. chaffeensis*-containing inclusions.

### B7 blocks Etf-1 localization to *E. chaffeensis*-inclusion membranes

Several Etf-1-binding peptides, including B7, C8, B7-16, B7-95-2, B7-131-1, B7-131-5, B7-133-3, and B7-133-8, significantly inhibited *E. chaffeensis* infection of THP-1 cells (Figs. 5, S3, and S4), we therefore investigated whether these peptides interfere Etf-1 localizations and functions. Our recent study showed that Etf-1-specific nanobodies blocks mitochondrial localization of Etf-1 and inhibit *E. chaffeensis* infection (18). Since B7 colocalizes with Etf-1, we examined whether prolonged treatment of B7 interferes with cellular localization of Etf-1. RF/6A cells ectopically expressing Etf-1-GFP protein were treated with 10 μM B7, control peptide C17, or vehicle control (DMSO) at 16 h post transfection (pt) and incubated for 2 days. Ectopically expressed Etf-1-GFP localizes to filamentous-shaped mitochondria in RF/6A cells in the control group (Fig. S7) as demonstrated previously (16, 18). However, unlike Etf-1-specific neutralizing nanobody that blocked mitochondrial localization of Etf-1–GFP (18), neither peptide B7 nor C17 affected mitochondrial localization of Etf-1-GFP protein (Fig. S7).

**Fig. 7.**
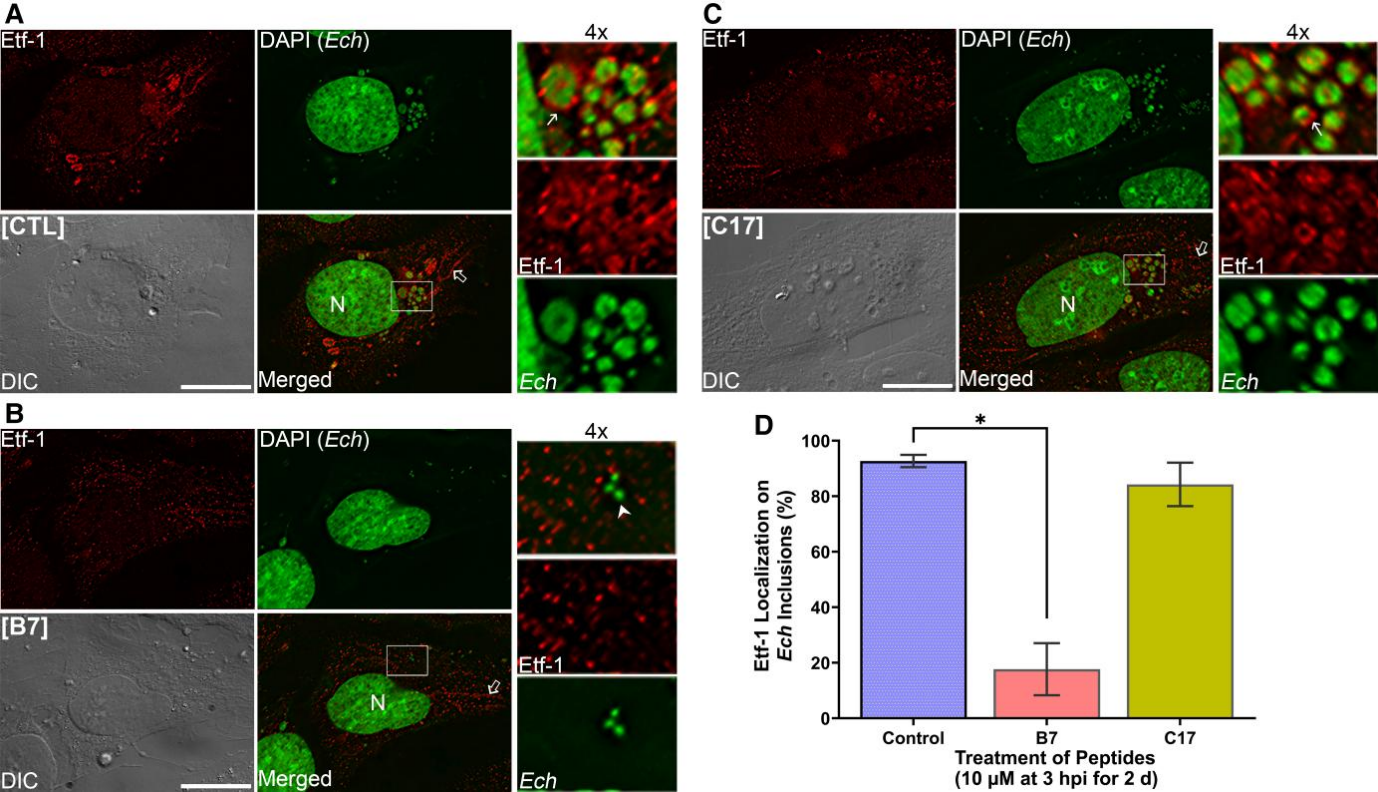
Macrocyclic peptide B7 abrogated Etf-1 localization on *E. chaffeensis* inclusions. RF/6A cells were seeded onto a coverglass in a 12-well plate for 1 h, and infected with *E. chaffeensis* (*Ech*) at ∼30 MOI. At 3 h pi, cells were treated with 10 μM B7, C17, or DMSO control (CTL), and remained in the culture for 2 days. Cells were fixed in 4% PFA and labeled with rabbit anti-Etf-1 IgG and AF488-conjugated goat anti-rabbit secondary antibody in PGS for 1 h each at RT. DAPI was used to label both host-cell nuclei and *Ehrlichia* DNAs (pseudocolored in green), and *E. chaffeensis* organisms were shown as green-colored, round to pleomorphic cocci (arrowhead in panel B) that further clustered as spherical morulae. Thin arrows, *E. chaffeensis*-containing inclusion; open arrows, filamentous-shaped mitochondria. DeltaVision microscope. (A–C) Images were the representative of at least three independent experiments. Bar, 10 μm. (D) Percentage of *E. chaffeensis* inclusions colocalized with Etf-1 were quantified by counting ∼20 cells per group from three independent experiments. Results are shown as the mean ± SD. *Significantly different from the control group (*P* < 0.01, ANOVA).

Etf-1 can also induce RAB5-regulated autophagosomes and fuse with *E. chaffeensis*-containing inclusions to deliver host nutrients (15, 34). We therefore investigated whether prolonged treatment of B7 can prevent the localization of Etf-1 to bacterial inclusions in infected cells. Immunofluorescence labeling showed that, similar to our previous findings (15, 16), native Etf-1 protein was localized to *E. chaffeensis* inclusions (Fig. 7A and C, thin arrows in enlarged panels) as well as mitochondria (Fig. 7A–C, open arrows in merged images) in *E. chaffeensis*-infected RF/6A cells treated with DMSO or peptide C17. However, B7 treatment significantly inhibited the localization of Etf-1 to *E. chaffeensis* inclusions (Fig. 7B and D, *E. chaffeensis* was indicated by arrowheads in panel B), as well as *E. chaffeensis* infection in RF/6A cells similar to that in THP-1 cells (Fig. S4), which was demonstrated by reduction of the numbers and sizes of *E. chaffeensis*-containing morulae compared to the control or C17-treated groups (Fig. 7A–C). B7 did not affect the mitochondrial localization of native Etf-1 protein secreted by *E. chaffeensis* (Fig. 7B, open arrow), similar to that of ectopically expressed Etf-1 protein (Fig. S7).

### Etf-1-binding peptides disrupt direct binding of Etf-1 to Beclin-1

**Fig. 8.**
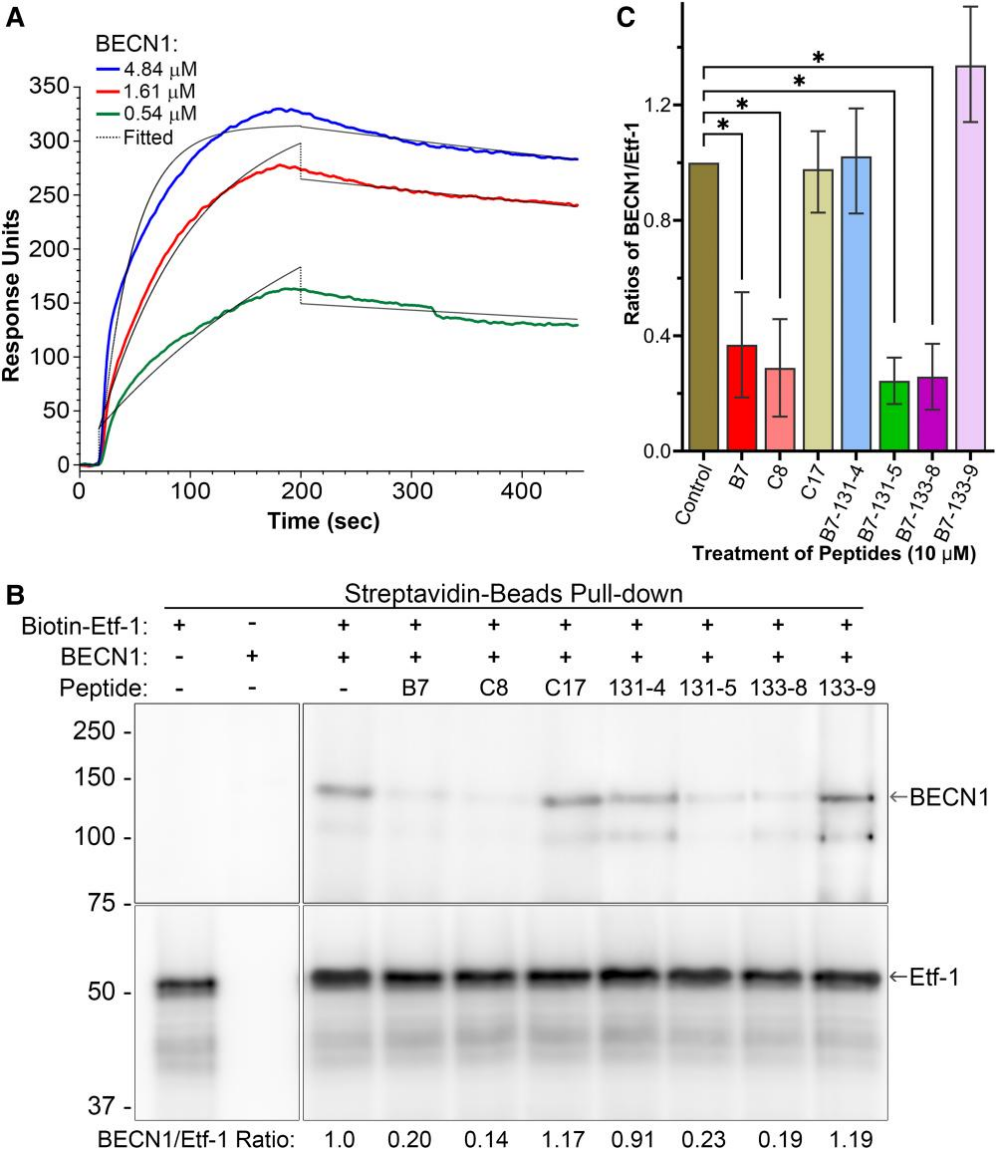
Determination of binding affinity of BECN1 to Etf-1 and disruption of BECN1/Etf-1 binding by macrocyclic peptides. (A) Binding affinity of BECN1 to Etf-1 was determined by OpenSPR. A series of three-fold dilutions of recombinant full-length BECN1 protein was used to test their binding against biotinylated Etf-1 (1 μM) immobilized on a sensor chip. Colored lines are signals detected by OpenSPR and thin black lines are fitted models generated by the TraceDrawer software. Binding affinity (*K*_D_) of BECN1 to Etf-1 is calculated as 60 ± 1 nM. (B and C) Co-precipitation of BECN1 with biotin-labeled Etf-1 using streptavidin beads pull-down assay. Streptavidin magnetic beads were incubated with biotinylated full-length Etf-1 protein (Biotin-Etf-1) at 4°C overnight, then incubated with 10 µM of Etf-1-binding macrocyclic peptides or DMSO control (−) for 2 h at RT. Approximate equal molar concentration of Beclin 1 protein (BECN1) was added to Etf-1/peptide complex and incubated for additional 3 h at RT. Proteins were eluted with 50 µL of 1× SDS-sample buffer, and subjected to Western blotting analyses with antibodies against Etf-1 and BECN1 (B). Band intensities were quantitated by ImageQuantTL, and relative ratios of BECN1/Etf-1 were calculated with the ratio of DMSO control group set as 1. The results were plotted as the mean ± standard deviation from three independent experiments and statistical analysis was performed using GraphPad Prism (C). *Significantly different from the control group (*P* < 0.01, ANOVA).

Our previous co-immunoprecipitation study showed that Etf-1 interacts with human RAB5, BECN1, and VPS34 complex (15); However, whether Etf-1 can directly bind to BECN1 was not determined. We, therefore, performed open surface plasmon resonance (OpenSPR) and in vitro pull-down assays to determine the direct PPI between Etf-1 and BECN1, and whether it is disrupted by Etf-1-binding peptides. By immobilizing biotinylated rEtf-1 proteins on a streptavidin-sensor chip and using recombinant full-length BECN1 protein as analyte, the OpenSPR data showed that Etf-1 bound to BECN1 with a dissociation constant (*K*_D_) of 60 ± 1 nM (Fig. 8A). However, due to strong nonspecific binding of the peptides and unlabeled rEtf-1 to the streptavidin-sensor chip, we are unable to determine the effects of the peptides on the binding of Etf-1 to BECN1 by OpenSPR. We therefore employed a biotinylated Etf-1 protein immobilized on streptavidin-beads to pull-down BECN1 in vitro. As shown in Fig. 8B, BECN1 protein were co-precipitated and eluted with Etf-1 protein, confirming the direct binding of Etf-1 to BECN1. By Western blotting and quantitation of the band intensities of BECN1 bound to Etf-1 in the presence of the peptides, we showed that B7, C8, 131-5, and 133-8, but not C17, 131-4, and 133-9, disrupted BECN1 binding to Etf-1 compared to the DMSO control (Fig. 8B and C). These data are consistent with the effects of these peptides on inhibiting *E. chaffeensis* infection (Fig. 5), suggesting a strong correlation of inhibiting bacterial infection and blocking Etf-1–BECN1 interaction by Etf-1-binding peptides.

## Discussion

Infections caused by intracellular bacterial pathogens are difficult to treat because a drug molecule must first travel across the plasma membrane of the host cell and then the membrane(s) of the bacterial pathogen if the intended target is localized inside the bacterium. For bacterial pathogens that rely on secreted proteins for infection and/or survival, the secreted proteins represent attractive targets for antibacterial drug design because a drug molecule only needs to cross the host cell membrane to reach the target. Since the obligatory intracellular bacterium *E. chaffeensis* requires a secreted effector protein Etf-1 for host cell infection, inhibitors against the Etf-1 protein may provide effective treatments of *E. chaffeensis* infections. Indeed, we previously demonstrated that an Etf-1-binding nanobody (D7) blocks the mitochondrial localization of Etf-1 and inhibits mitochondria-mediated apoptosis, thereby reduces *E. chaffeensis* infection in mammalian cells and in a mouse model (18). However, clinical development of the cyclic CPP-nanobody conjugate is challenging because of its high cost of production and the site-specific conjugation of a cyclic CPP to the nanobody. Development of small-molecule inhibitors against Etf-1 is likely to be difficult as well, because Etf-1 functions by engaging in intracellular PPIs and may not contain suitable pockets for small molecules to bind. The absence of a three-dimensional structure for Etf-1 further complicates any small-molecule inhibitor design.

Macrocyclic peptides represent an emerging drug modality, which are capable of binding to flat protein surfaces (e.g. PPI interfaces) with antibody-like affinity and specificity. By integrating macrocyclic peptides and cyclic CPPs, we have previously generated cell-permeable, metabolically stable, potent, and specific inhibitors against a variety of intracellular PPI targets in mammalian cells (19, 20, 30, 36, 37). However, to our knowledge, macrocyclic peptides have not been used to treat infections caused by intracellular bacterial pathogens. In this study, we took advantage of the fact that *E. chaffeensis* infection requires secreted Etf-1 protein in host cytosol, and discovered cell-permeable macrocyclic peptides to specifically block the function of Etf-1.

The macrocyclic peptide library is designed to contain a B-ring for cell entry, while both A- and B-rings contribute to target binding. Initial screening of a macrocyclic peptide library identified peptides B7 and C8 as moderately potent Etf-1 binders (low μM *K*_D_) that are cell permeable and inhibit host cell infection by *E. chaffeensis*. Subsequent optimization of B7 improved its Etf-1-binding affinity by 2- to 10-fold, resulting in several potent Etf-1 ligands (e.g. IC_50_ = 110 nM for B7-133-8). Surprisingly, despite a greater sequence diversity in the A-ring, residues in the B-ring are more critical for Etf-1 binding based on alanine-scan data. Since arginine, phenylalanine, and tryptophan (for which naphthylalanine is an analog) are some of the most common “hot” residues for PPIs, their presence in the B-ring renders the B-ring prone to protein binding. Nevertheless, the A ring also makes important contributions to Etf-1 binding, as reflected by the alanine-scan data (e.g. Tyr and Leu) as well as the improved binders from the second-generation library screening efforts, which involved mostly modifications of the A-ring. Accordingly, the improved peptides (e.g. B7-133-3 and B7-133-8) were significantly more potent than B7 in inhibiting *Ehrlichia* infection. Unfortunately, the improved Etf-1 inhibitors also showed greater cytotoxicity in mammalian cells and further medicinal chemistry efforts are necessary to reduce the off-targeting binding and cytotoxicity.

It is worth noting that many of the peptides showed *K*_D_ values for Etf-1 that were much greater than the corresponding IC_50_ values in the FP-based competition assay. This is unusual and likely caused by an overestimation of the *K*_D_ values. The simplest explanation is that only a fraction of the recombinant Etf-1 protein produced in *Escherichia coli* was properly folded and biologically active; while this results in an overestimate of the biologically active Etf-1 concentration (and thus the *K*_D_ value) in the FP-based direct binding assay, it has much less effect on the FP-based competition assay. This scenario is consistent with the observation that despite of their μM *K*_D_ values for Etf-1, many of the peptides effectively disrupted the tight Etf-1/BECN1 interaction (*K*_D_ = 60 nM) in both biochemical and cellular assays.

In addition to providing a potential treatment of the tick-born disease, the macrocyclic peptidyl Etf-1 inhibitors have served as useful chemical probes to gain significant insight into the molecular mechanism of *E. chaffeensis* infection. We showed that Etf-1 binds directly to BECN1 with high affinity (*K*_D_ = 60 nM) and peptide B7 and its derivatives disrupt the Etf-1/BECN1 interaction inside the cytosol of host cells. Etf-1–induced autophagosomes, which possess features of early endosomes and early autophagosomes like RAB5, BECN1, and VPS34, and capture host-cell cytoplasmic nutrients (e.g. amino acids), are trafficked to and fused with pre-established *E. chaffeensis* inclusions for continuous *E. chaffeensis* replication and expansion of the inclusions (15). Our findings suggest a strong correlation of the direct binding of Etf-1 to BECN1 in supporting *Ehrlichia* infection. Disruption of the Etf-1/BECN1 interaction by the macrocyclic peptides likely blocks the nutrient delivery into *E. chaffeensis* inclusions and, therefore, inhibits bacterial proliferation. Nevertheless, how these Etf-1-binding peptides affect interaction/activation of RAB5-PIK3C3 complex as well as autophagosome formation remains to be studied.

Other than inducing autophagosome formation, a subset of Etf-1 is translocated to mitochondria and inhibits cellular apoptosis, allowing sufficient time for *E. chaffeensis* to replicate (16). Interestingly, although the macrocyclic peptides colocalized with Etf-1 on the mitochondria, they did not block Etf-1 mitochondrial localization. On the other hand, we previously showed that a nanobody against Etf-1 blocked the mitochondrial localization of Etf-1 and inhibited mitochondria-mediated apoptosis of host cells, thereby reducing *E. chaffeensis* in mammalian cells and in a mouse model (18). These observations suggest that the macrocyclic peptides in this work and the nanobodies in our earlier study likely bind to distinct sites on Etf-1 (18). It will be interesting to test whether the macrocyclic peptides and nanobody D7 act synergistically to inhibit *E. chaffeensis* infection.

Etf-1 homologs are found in all sequenced members of the genera *Ehrlichia* and *Anaplasma* (Ats-1 in *A. phagocytophilum*), which are human and/or animal pathogens (18). We have previously shown that Ats-1 also directly binds BECN1 (38). Our studies suggest that the mechanisms and functions of this T4SS effector are likely conserved among the genera *Ehrlichia* and *Anaplasma.* Thus, the macrocyclic peptide approach demonstrated in this work may be applicable to other members of the *Ehrlichia* and *Anaplasma* genera. Additionally, cell-permeable macrocyclic peptidyl inhibitors may be developed against other bacterial virulence proteins, binding ligands/receptors, and/or signaling pathways. In conclusion, the present study has demonstrated the feasibility of developing cell-permeable macrocyclic peptides as a novel class of antibacterial agents against intracellular bacterial pathogens.

## Materials and methods

### Reagents and antibodies

Reagents for peptide synthesis were purchased from Chem-Impex (Wood Dale, IL, USA), NovaBiochem (La Jolla, CA, USA), or Anaspec (San Jose, CA, USA). Trimesic acid, fluorescein isothiocyanate isomer I, 5(6)-carboxyfluorescein and tetrakis(triphenylphosphine)palladium (0) [Pd(PPh_3_)_4_] were purchased from Sigma-Aldrich (St Louis, MO, USA). 5(6)-Carboxynaphthofluorescein and 5(6)-carboxynaphthofluorescein succinimidyl ester were purchased from Setareh Biotech (Eugene, OR, USA). Phenylsilane was purchased from TCI America (Portland, OR, USA). All solvents and other chemical reagents were obtained from Sigma-Aldrich, Fisher Scientific (Pittsburgh, PA, USA), or VWR (West Chester, PA, USA) and were used without further purification. LC-MS grades solvents (Optima LC/MS Grade) were purchased from Fisher Scientific. Cell culture media, heat-inactivated fetal bovine serum (FBS), penicillin-streptomycin (Pen-Strep), 0.25% trypsin-EDTA, Dulbecco's modified phosphate-buffered saline (DPBS) and protease inhibitor cocktail were purchased from Sigma-Aldrich. NHS-PEG4-D-Biotin was purchased from Life Technologies (Carlsbad, CA, USA).

The following antibodies were used in this study: affinity-purified rabbit IgG against the C-terminal 250 aa of Etf-1 (residues 131–380) (16); rabbit anti-*E. chaffeensis* recombinant major outer membrane proteins P28 (39); HisProbe-horseradish peroxidase (HRP)–conjugated (Thermo Fisher Scientific, Waltham, MA, USA); rabbit anti-actin (Sigma-Aldrich); mouse monoclonal anti-cytochrome *C* (Santa Cruz Biotechnology, Dallas, TX, USA); Alexa Fluor (AF) 488- or AF555-conjugated goat anti-mouse IgG and anti-rabbit IgG (Invitrogen, Carlsbad, CA, USA), HRP-conjugated goat anti-mouse IgG and anti-rabbit IgG (Cell Signaling Technology), and HRP-conjugated streptavidin (Thermo Fisher Scientific).

### Recombinant protein expression, purification, and biotinylation

The coding sequence for full-length Etf-1 containing an N-terminal 6×His-tag was cloned into pET33b(+) vector to generate a plasmid expressing 6×His-Etf-1 fusion protein. Recombinant Etf-1 protein was expressed in *E. coli* BL21(DE3) (New England Biolabs, Ipswich, MA, USA) and purified by affinity chromatography using HisPur Cobalt resin (Thermo Scientific, Rockford, IL, USA) as previously described (40). Human full-length *BECN1* gene was optimized for expression in *E. coli*, cloned into a pET-21b vector, and recombinant BECN1 protein was expressed and purified as described previous (41). Recombinant proteins were further purified by size exclusion chromatography on an AKTA Purifier 10 FPLC system (GE Healthcare, Piscataway, NJ, USA) equipped with a Superdex 200 Increase 10/300 GL column (GE Healthcare). The proteins were concentrated to 1.5–2.2 mg/mL and exchanged into PBS (pH 7.4) containing 2 mM DTT and 10% glycerol in an Amicon Ultra-15 centrifugal concentrator (30 K MWCO, EMD Millipore, Burlington, MA, USA). Etf-1 was biotinylated by mixing with two equivalents of NHS-PEG4-D-Biotin in PBS buffer. The pH of the solution was adjusted to 8.0 by using 50 mM sodium bicarbonate (pH 8.0) and the reaction was allowed to proceed for 2 h at 4°C. The reaction was quenched by the addition of 100 mM Tris buffer, pH 7.4, and the biotinylated Etf-1 was stored at 4°C for peptide screening and PPI.

### Peptide synthesis and purification

Peptides were manually synthesized on Rink amide resin LS (100–200 mesh, 0.43 mmol/g, 50–100 mg scale) using standard Fmoc chemistry. The typical coupling reaction contained 5 equivalents of Fmoc-amino acid, 5 equivalents of azabenzotriazole tetramethyl uronium hexafluorophosphate (HATU), and 10 equivalents of diisopropylethylamine (DIPEA) in *N*,*N*-dimethylformamide (DMF) and dichloromethane (DCM) (80:20 v/v). The reaction was proceeded with mixing for 40 min at room temperature. Following the synthesis of the linear sequence, trimesic acid (TMA) was coupled to the N-terminus using 10 equivalents of TMA, 3 equivalents of HATU, and 30 equivalents of DIPEA in DMF/DCM mixture. Next, the Alloc groups from the Dap side chains were removed using 0.3 equivalent of Pd(PPh_3_)_4_ and 10 equivalents of phenylsilane in DCM (dark, 3 × 20 min). Then the peptides were cyclized on-resin using 10 equivalents of benzotriazol-1-yloxytripyrrolidinophosphonium hexafluorophosphate (PyBOP), 10 equivalents of Hydroxybenzotriazole (HOBt), and 20 equivalents of DIPEA in DMF/DCM (2 × 90 min). Peptides were cleaved and deprotected with 92.5% (v/v) trifluoroacetic acid (TFA), 2.5% (v/v) water, 2.5% (v/v) dimethoxybenzene and 2.5% (v/v) triisopropylsilane for 3 h. The solvents were removed by flowing a stream of N_2_ over the crude peptide solution, following which the peptide residue was triturated with cold diethyl ether (three times). The crude peptides were purified by reversed-phase HPLC equipped with a preparative Phenomenex C_18_ column (5 μm, 21.2 mm I.D., 250 mm length), eluting with linear gradients of acetonitrile in ddH_2_O (each containing 0.05% TFA). For biotinylated peptides, D-biotin was conjugated to the C-terminal lysine side chain of the pure peptide using 1 equivalent of EZ-Link NHS-PEG4-D-Biotin in solution phase (PBS buffer, pH 8.5) and purified with reversed-phase HPLC. For the FAM-labeled peptides, fluorescein was conjugated to the C-terminus lysine residue of the peptides on resin by using 5(6)-carboxyfluorescein or in solution by using 5′-FITC. The purity of the peptides (>95%) was assessed by reversed-phase UPLC equipped with an analytical Waters Acquity UPLC BEH C18 column (1.7 μm, 2.1 mm I.D., 100 mm length) and an inline UV-Vis detector. The authenticity of each peptide was confirmed by high-resolution mass spectrometry (HR-MS) using a Bruker 15 T MALDI-FT-ICR instrument.

### Cell-permeable macrocyclic peptide library screening

For each screening experiment, ∼400 mg of the macrocyclic peptide library was swollen in DCM and washed extensively with DMF, ddH_2_O and finally incubated overnight at 4°C in 10 mL of blocking buffer (30 mM sodium phosphate, pH 7.4, 150 mM NaCl, 0.05% Tween 20, 3% BSA and 0.1% gelatin). The solution was drained, and the resin was resuspended in 10 mL of blocking buffer containing 500 nM biotinylated Etf-1 for overnight at 4°C. The resin was washed with blocking buffer and resuspended in 10 mL of blocking buffer. M280 streptavidin-coated Dynabeads (Invitrogen; 20 μL) were added to the resin and the mixture was incubated on a rotary wheel for 1 h at 4°C. The magnetic beads were isolated from the bulk by using a TA Dynal MPC-1 magnetic particle concentrator (Invitrogen). The beads were transferred to a Bio-Spin column (0.8 mL; BioRad, Hercules, CA, USA) and incubated in blocking buffer containing 100 nM biotinylated Etf-1 for 4 h at 4°C. The solution was drained, and the resin was quickly washed with blocking buffer to remove any unbound protein. The resin was resuspended in 1 mL of blocking buffer and streptavidin-alkaline phosphatase (SA-AP) conjugate was added to the mixture (1 μg/mL final concentration). After 10 min at 4°C the solution was drained and the beads were quickly washed with 1 mL of blocking buffer (3×) and 1 mL of staining buffer (30 mM Tris, pH 8.5, 100 mM NaCl, 5 mM MgCl_2_, and 20 μM ZnCl_2_) (3×). The resin was resuspended in 1.5 mL of staining buffer in a Petri dish and 150 μL of a 5-bromo-4-chloro-3-indolyl-phosphate (BCIP) solution (5 mg/mL) was added. After 30 min, 100 μL of 1 M HCl was added to quench the reaction and the intensely turquoise positive beads were manually isolated under a dissecting microscope. Peptide sequences of hit beads were determined using partial Edman degradation-mass spectrometry (PED-MS) as previously described (25, 37).

### Synthesis of the focused (second generation) peptide library

The invariant C-terminal sequence (D-Leu-Dap-4-ClPhe- D-Nal- D-Arg-Arg- D-Arg-Arg-Dap-β-Ala-β-Ala-Lys-Met) was synthesized on 2 g of TentaGel resin (90 μm, 0.29 mmol/g loading) using the standard Fmoc/HATU peptide synthesis protocol. After removal of the N-terminal Fmoc group from D-Leu, the resin was split into 29 equal aliquots and placed into 29 different micro-spin columns, and a different Fmoc-amino acid (5 equivalents) was coupled to each aliquot of resin. Each coupling reaction was supplemented with 10 mol% of acetic acid (relative to Fmoc-amino acid). In addition, 5 mol% deuterated acetic acid (CD_3_CO_2_D was added to the coupling reactions of L-Ala, cis-AcPc, D-Leu, L-Orn, L-isoAsp, D-Lys, and D-Tyr), while 5 mol% deuterated propanoic acid (CH_3_CD_2_CO_2_H) was added to L-Nle and β-Ala reactions. The inclusion of the carboxylic acids generates a series of sequence-specific peptide truncation products, which facilitate the later sequence determination by mass spectrometry (42). After the coupling reactions were complete, the resin from all 29 columns was pooled together and the N-terminal Fmoc group was removed by piperidine. Subsequently, the split-and-pool protocol was repeated to install the other randomized residues. To vary the length of the random region, a small portion of the resin was removed from the library and set aside after the coupling of each randomized residue. After all five randomized residues were added, all resin aliquots (including the portions set aside) were pooled and treated with 20% piperidine to remove the N-terminal Fmoc group. The invariant tyrosine residue (along with 5 mol% CH_3_CD_2_CO_2_H) was then coupled to the peptide. Next, the resin was treated with TMA/HATU/DIPEA (10, 3, and 20 equivalents, respectively) for 1 h. The Alloc protecting groups on the Dap residues were removed by incubating the resin (three times) with 0.5 equivalent of tetrakis(triphenylphosphine)palladium and 10 equivalents of phenylsilane for 20 min. The resin was washed with 2% sodium dimethyldithiocarbamate in DMF to remove the Pd(0) reagent and then 1 M HOBt in DMF. Finally, the peptides were cyclized by treatment for 1 h (twice) with PyBOP/HOBt/DIPEA (10, 10, and 20 equivalents, respectively). The peptides were deprotected by treating the resin for 4 h with a modified reagent K (2.5% triisopropylsilane, 2.5% H_2_O, 2.5% 2,2′-(ethylenedioxy)diethanethiol, 2.5% phenol, in TFA). The library was dried under vacuum and stored in −20°C until use.

### Etf-1 ligand binding assay and FP-based competition assay

Etf-1 and macrocyclic peptides were serially diluted in PBS supplemented with 5 mM DTT and 0.01% Triton X-100 (v/v). FAM-labeled peptides (50 nM) were prepared from a 10-μM DMSO stock and incubated with serially diluted Etf-1 for 1 h at room temperature (RT). Twenty microliters of the solutions were transferred to a black 384-well plate (Greiner Bio-One) and FP values were measured on a Tecan Infinite M1000 Pro plate reader with excitation and emission wavelengths at 470 and 535 nm, respectively. Titration curves (*n* ≥ 3) were fitted using GraphPad Prism v8.0 (GraphPad, La Jolla, CA, USA), and analyzed with TraceDrawer software v1.8 (Ridgeview Instruments AB, Uppsala, Sweden) to calculate the dissociation constant (*K*_D_, in nM).

For competitive binding experiments, FAM-labeled probe (FAM-B7-133-8; 50 nM) was incubated with Etf-1 (500 nM) in PBS (pH 7.4) containing 2 mM TCEP for 1 h. Serial dilutions of competitor peptide (0-20 μM) were prepared from DMSO stock solutions into PBS containing 0.01% Triton-X100 and 2 mM TCEP and added to aliquots of the equilibrated Etf-1/probe solution. After incubation for 1 h in the dark and with gentle mixing, 20 μL of each sample was transferred into 384-well black-on-black microplates and FP was measured using a TECAN Infinite M1000 plate reader. Data were analyzed using GraphPad PRISM ver. 8.0 and fit to a four-parameter variable slope equation to determine IC_50_ values. IC_50_ values reported are the mean ± SD of *n* = 2–4 independent experiments.

### Confocal microscopy

HeLa cells (15,000 cells) were seeded in 300 μL of growth media (DMEM, 10% FBS, 1% Pen-Strep) per well in a 35-mm glass-bottomed microwell dish with 4 compartments (Greiner) and cultured overnight. Next day, the cells were gently washed with DPBS twice, and treated for 2 h with 5 μM FAM-labelled peptides in phenol red-free DMEM containing 1% and 1% Pen-Strep. After removal of the medium, the cells were gently washed with DPBS twice and imaged on a Nikon A1R live-cell confocal laser scanning (ECLIPSE Ti-E automated, inverted) microscope equipped with a 100× oil objective (1.45 N.A.) and a heated (37°C) chamber supplied with 5% CO_2_. The data were analyzed using NIS-Elements AR.

### Flow cytometry

HeLa cells were seeded in a 12-well plate at a density 1.5 × 10^5^ cells per well in 1 mL of growth media and cultured overnight. Next day, NF-labeled peptide was added to a final concentration of 2 or 5 μM in DMEM supplemented with 10% FBS and 1% Pen-Strep and incubated at 37°C for 2 h. After incubation, the cells were washed with cold DPBS twice, detached from the plate with 0.25% trypsin, diluted into cold DPBS, and pelleted at 300*g* for 5 min at 4°C. The supernatant was discarded, the cells were washed twice with cold DPBS and resuspended in 200 μL of cold DPBS. The samples were analyzed on a BD FACS LSR II flow cytometer. For NF-labelled peptides, a 633-nm laser was used for excitation and the fluorescence emission was analyzed in the APC channel. MFI was determined and the histogram plots were generated in FlowJo software.

### Cell viability and LDH assay

Cytotoxicity of Etf-1-binding peptides was examined by CellTiter-Glo luminescent cell viability assay (Promega, Madison, WI, USA). THP-1 cells were seeded in a transparent 96-well plate at a density of 5 × 10^3^ cells/well (100 μL in each well) in RPMI-1640 containing 10% FBS and 1% antibiotics and cultured overnight. Etf-1-binding peptides were serially diluted in DPBS from a DMSO stock and added to the cells with the final concentration of 0–25 μM and a constant final concentration of 0.5% DMSO (v/v). After incubation at 37°C with 5% CO_2_ for 46 h, the plate was removed and pre-equilibrated to RT before the addition of 100 μL of the CellTiter-Glo 2.0 reagent to each well. The plate was incubated for 15 min on a rotary shaker in the dark, and the luminescence was detected (1-s integration time) on a Tecan Infinite M1000 Pro microplate reader. Relative luminescence was plotted as function of the peptide concentration, with the cell viability of DMSO control group set as 100%.

LDH release assay (Promega) was performed to assess the level of plasma membrane damage in a cell population by these compounds. Peptides were serially diluted in DPBS and added to each well with a constant final concentration of 0.5% DMSO (v/v). Control wells contained 10 μL of lysis buffer, cell-free complete growth media, or positive LDH control. Following incubation at 37°C and 5% CO_2_ for 45 to 75 min, the culture was centrifuged and 50 μL of the growth medium was withdrawn from each well, transferred to a clear 96-well plate, mixed with 50 μL of LDH substrate mixture, and incubated at RT for 30 min with gentle mixing. Finally, 50 μL of 1 N HCl was added to each well, and the absorbances at 490 and 680 nm were immediately measured on a Tecan Infinite M1000 plate reader. Relative membrane leakage was calculated and plotted using GraphPad Prism v8.0.

### Serum stability assay

Human serum was diluted to 25% (v/v) in sterile DPBS and equilibrated at 37°C for 15 min. Peptides were added to the diluted serum to a final concentration of 20 μM and incubated at 37°C with gentle mixing. At designated time points, 30 μL aliquots were withdrawn and mixed with 30 μL of 15% trichloroacetic acid in MeOH (w/v) and 30 μL of MeCN and stored at 4°C overnight to completely precipitate the serum proteins. Next, each aliquot was centrifuged (15,000*g*, 5 min, 4°C) and 14 μL of the supernatant was injected in an UPLC equipped with an analytical Waters Acquity UPLC BEH C18 column (1.7 μm, 2.1 mm I.D., 100 mm length) and an inline UV-Vis detector and a single quadrupole mass detector. The column was eluted with a linear gradient of 20–50% MeCN in ddH_2_O containing 0.1% TFA. A UV/Vis detector at 214 nm and the mass spec quantifying the ion counts corresponding to the [M + 3H]^3+^ ion of the analytes were used to analyze the samples. The area under the peak of interest was obtained by integration, normalized to that of the peptide at *t* = 0, and plotted as a function of the incubation time to determine the serum half-life of the peptide.

### Analysis of macrocyclic peptides on *E. chaffeensis* infection and qPCR assay

*Ehrlichia chaffeensis* Arkansas strain (43) was cultured in THP-1 cells (ATCC, Manassas, VA, USA) in RPMI 1640 medium (Corning, Manassas, VA, USA) supplemented with 8% FBS (Atlanta Biologicals, Flowery Branch, GA, USA) and 4 mM L-glutamine (at 37°C under 5% CO_2_ in a humidified atmosphere. THP-1 cells were seeded at 1 × 10^5^ cells/well (in 1.5-mL RPMI 1640 culture medium with 8% FBS, 4 mM L-Gln) in a 12-well plate, then infected with host cell-free *E. chaffeensis* at an MOI (multiplicity of infection, or numbers of bacteria per cell) of ∼30. Cells were cultured in a humidified incubator with 5% CO_2_ and 95% air. No antibiotic was used throughout the study. At 0.5 h post infection (pi), cells were incubated with 1–10 μM of peptides or with an equal volume of DMSO (solvent control, 2 μL per mL medium). At 2 days pi, cells were washed with 1 mL of PBS, and two drops of cells were cytospun onto glass slides by a Shandon Cytospin 4 cytocentrifuge (Thermo Fisher Scientific), then fixed and stained with HEMA 3 staining solutions (Thermo Fisher Scientific). *Ehrlichia* infection was observed and images were captured by a SPOT RT digital camera (Diagnostic Instruments, Sterling Heights, MI, USA) coupled to a Nikon Eclipse E400 microscope (Nikon, Melville, NY, USA).

DNA was extracted from each sample using Qiagen blood mini kit according to the manufacturer's instructions (Qiagen, Valencia, CA, USA), and qPCR analysis was performed in an MX3000P qPCR instrument (Stratagene, San Diego, CA, USA) using SYBR Green Real-Time PCR Master Mix (Thermo Fisher Scientific) according to the manufacturer's instructions. Five-fold serial dilutions of the control group were used to generate a standard curve to calculate the relative copy numbers of *E. chaffeensis* 16S rRNA normalized against human actin genes using the MX3000P software from Stratagene as described previously (18, 44).

### Immunofluorescence labeling assay of cellular localization of Etf-1, macrocyclic peptides, and *E. chaffeensis*

To examine the cellular distribution of Etf-1 regulated by macrocyclic peptides, RF/6A cells (ATCC), which were cultured in Advanced Minimum Essential Medium (AMEM; Gibco, Grand Island, NY, USA) with 5% FBS and 4 mM L-glutamine, were used for immunofluorescence labeling assay and localization analysis. Plasmids encoding *E. chaffeensis* Etf-1 (codon-optimized for mammalian expression) fused with C-terminal GFP or DsRed tags were transformed into *E. coli* strain DH5α (Invitrogen) and purified using the Endo-Free Plasmid Purification kit (QIAGEN) (16).

For mitochondria localization analysis, RF/6A cells were seeded onto a cover glass in a 12-well plate for 3 h, then infected with *E. chaffeensis* at ∼30 MOI. Alternatively, cells were transfected with Etf-1-GFP or Etf-1-DsRed plasmids using Fugene HD (Promega) or by electroporation at 100 V and 1,000 µF using the Gene Pulser Xcell System (BioRad) according to the manufacturers’ instructions (16). Cells were treated with 10 μM peptide B7, C17, or DMSO control at 3 h pi, or FAM-labelled B7 or C17 at 2 h prior to fixation. To examine the roles of macrocyclic peptides on Etf-1 localization on *E. chaffeensis* inclusions, infected RF/6A cells were treated with 10 μM peptides at 3 h pi for 2 days. At 2 days pi or pt, cells were washed 3 times with PBS, then fixed in 4% PFA for 20 min. Native Etf-1 and mitochondria marker cytochrome C proteins were labeled with rabbit anti-Etf-1 IgG and mouse anti-cytochrome C, then with AF488-conjugated goat anti-rabbit and anti-mouse secondary antibodies in PGS (PBS with 0.1% gelatin and 0.1% saponin) for 1 h each at RT. Host-cell and Ehrlichial DNAs were stained with 300 nM DAPI, and fluorescence with differential interference contrast images were observed and captured with a DeltaVision deconvolution microscope (Applied Precision, Issaquah, WA, USA). Percentages of Etf-1 protein colocalization with macrocyclic peptides or *E. chaffeensis* inclusions were quantified by counting 10–20 cells per group from three independent experiments.

### Characterization of BECN1 binding to Etf-1 by OpenSPR

The equilibrium dissociation constant (*K*_D_ or affinity constant) of BECN1 and Etf-1 was measured by OpenSPR (Nicoya, Kitchener, ON, Canada). After immobilization of biotinylated rEtf-1 (1 μM) to a streptavidin-sensor chip (Nicoya), dilutions of BECN1 in Tris running buffer (20 mM Tris, pH 8.0, 100 mM NaCl, 0.05% Tween 20) as analysts were slowly flowed over the sensor chip at a rate of 30 µL/min with a contact time of 150 s and dissociation time of 300 s. Binding kinetics were calculated using TraceDrawer software.

### Streptavidin magnetic beads pull-down assay and Western blot analysis

Streptavidin magnetic beads (15 µL, Pierce, Rockford, IL, USA) were washed with 1 mL of Bind/Wash buffer (25 mM Tris, pH 7.4; 250 mM NaCl, 0.1% Tween 20), and incubated with 0.15 µg (5 µM final concentration) of biotinylated full-length Etf-1 protein in bind/wash buffer at 4°C overnight with end-to-end rotation. Beads were washed three times with Bind/Wash buffer, then incubated with Etf-1-binding macrocyclic peptides (10 µM) or DMSO control in Bind/Wash buffer at room temperature for 2 h. Approximate equal molar concentration of BECN1 protein (0.25 µg, or 5 µM final concentration) was added to each group containing Etf-1/peptide complexes and incubated for 3 h at room temperature. Beads were washed three times, and proteins were eluted from the beads with 50 µL of 2× SDS-sample buffer. Samples were boiled for 5 min and subjected to Western blotting analyses with rabbit antibodies against Etf-1 and BECN1. Reacting bands were visualized with enhanced chemiluminescence by incubating the membrane with LumiGLO chemiluminescent reagent (Thermo Fisher Scientific). Images were captured by Amersham Imager 680 (GE Healthcare) and densitometry analysis was performed using ImagQuaNT (GE Healthcare). In each specimen, band intensities were normalized against those of actin as the loading control, and the ratios of the treatment group to the control group were calculated with the value in the control groups arbitrarily set as 1.0.

### Statistical analysis

All statistical analyses were performed with a one-way ANOVA using Prism 8 software (GraphPad). *P* < 0.05 was considered to reflect a statistically significant difference.

## Supplementary Material

pgad017_Supplementary_Data

## Data Availability

All experimental data described in this study are included in the main text and Supplementary information.
