# Supplementary material for: Inhibition of Ehrlichia chaffeensis infection by cell-permeable macrocyclic peptides that bind type IV secretion effector Etf-1
Source: PNAS Nexus. 2023 Jan 27;2(2):pgad017. doi: 10.1093/pnasnexus/pgad017 (PMC9982066; doi:10.1093/pnasnexus/pgad017)
Supplement: pgad017_Supplementary_Data [file pgad017_supplementary_data.pdf]

Supplementary Information for:

Inhibition of *Ehrlichia chaffeensis* Infection by Cell-Permeable Macrocyclic Peptides that Bind Type IV Secretion Effector Etf-1

Mingqun Lin [1 †], Amritendu Koley [2 †], Wenqing Zhang [1], Dehua Pei [2 *], and Yasuko Rikihisa [1 *]

[1] Department of Veterinary Biosciences, 1925 Coffey Rd;

[2] Department of Chemistry and Biochemistry, 484 West 12th Avenue;

The Ohio State University, Columbus, OH 43210

[†] Contributed equally

[*] Correspondences: Yasuko Rikihisa (rikihisa.1@osu.edu) or Dehua Pei (pei.3@osu.edu)

This PDF file includes:

- Table S1 to S3
- Figures S1 to S7

SUPPLEMENTARY TABLES

Supplementary Table S1: Sequences and Etf-1-binding affinities of macrocyclic peptides obtained from the first-round library screening

Peptide ID	Sequences [1]	K_D (μM) [2]
**B4**	TMA-His-Fpa-pro-Dap-Phe-Nal-Arg-Arg-Arg-Dap	ND
**B5**	TMA-Nle-Ala-leu-Dap-phe-Nal-Arg-arg-Arg-Dap	ND
**B7**	TMA-Tyr-His-Gln-pro-Ser-leu-Dap-Phe-nal-arg-Arg-arg-Arg-Dap	6.5 ± 0.7
**B8**	TMA-glu-thr-Ile-Dap-Nal-Arg-Arg-Arg-Phe-Dap	ND
**B9**	TMA-Orn-thr-Asp-Asp-Nle-Dap-Phe-Arg-Arg-Arg-Arg-Nal-Dap	ND
**B11**	TMA-Orn-Asp-Phg-His-val-Dap-phe-Nal-Arg-arg-Arg-arg-Dap	ND
**B12**	TMA-glu-Gln-Gln-glu-Dap-Phe-nal-arg-Arg-arg-Arg-Dap	ND
**B13**	TMA-glu-His-phe-glu-His-Ala-Dap-phe-Nal-Arg-arg-Arg-Dap	>25
**B14**	TMA-Phg-ala-His-Dap-Phe-Nal-Arg-Arg-Arg-Dap	ND
**B16**	TMA-Trp-nal-asn-Ser-Dap-Nal-Arg-Arg-Arg-Phe-Dap	ND
**B17**	TMA-Phg-phe-Gly-Asp-Tyr-Dap-Phe-nal-arg-Arg-arg-Arg-Dap	>25
**B19**	TMA-pro-Gln-val-Dap-phe-Nal-Arg-arg-Arg-arg-Dap	ND
**B20**	TMA-thr-Orn-Asp-nal-Dap-Phe-Nal-Arg-Arg-Arg-Arg-Arg-Dap	ND
**B21**	TMA-leu-His-Tyr-phe-Dap-arg-Arg-arg-Arg-Nal-Phe-Dap	ND
**B22**	TMA-val-Orn-asn-His-ala-Dap-Phe-nal-arg-Arg-arg-Arg-Dap	>25
**B23**	TMA-Asp-phe-val-Dap-Phe-Nal-Arg-Arg-Arg-Dap	ND
**C3**	TMA-Ile-nal-Gln-nal-Dap-arg-Arg-arg-Arg-Nal-Phe-Dap	ND
**C4**	TMA-His-asn-Ser-Dap-Phe-Nal-Arg-Arg-Arg-Dap	ND
**C5**	TMA-Ile-Als-Asp-Dap-phe-Nal-Arg-arg-Arg-Dap	ND
**C6**	TMA-Gln-His-Ser-val-Dap-phe-Nal-Arg-arg-Arg-Dap	>25
**C7**	TMA-lys-leu-Tyr-Dap-phe-Nal-Arg-arg-Arg-Dap	ND
**C8**	TMA-glu-val-Tyr-Trp-Gly-Dap-Phe-nal-arg-Arg-arg-Arg-Dap	5.0 ± 1.2
**C9**	TMA-Ile-Asp-Ala-Nle-His-Dap-Phe-Nal-Arg-Arg-Arg-Dap	>25
**C14**	TMA-Nle-Ile-glu-Dap-Arg-Arg-Arg-Nal-Phe-Dap	ND
**C15**	TMA-Gln-Gly-Phg-Dap-Phe-nal-arg-Arg-arg-Arg-Dap	ND
**C17**	TMA-glu-Ser-ala-phe-His-Ser-Dap-phe-Nal-Arg-arg-Arg-arg-Dap	>25
**C18**	TMA-Fpa-leu-nal-Dap-Arg-Arg-Phe-Arg-Nal-Arg-Dap	ND
**C19**	TMA-Ile-His-Ala-Orn-lys-Dap-Phe-Nal-Arg-Arg-Arg-Dap	>25
**C20**	TMA-Ser-pro-Fpa-Ile-Ser-Dap-Nal-Arg-Arg-Arg-Phe-Dap	ND
**C22**	TMA-leu-Gly-Ser-val-Ala-Dap-Nal-Arg-Arg-Arg-Phe-Dap	ND

[1] Abbreviations: Three-letter abbreviations with capitalized first letter are used for L-amino acids, while three-letter abbreviations in lowercase letters for D-amino acids. TMA, trimesic acid; Dap, 2,3-diaminopropionic acid; Fpa, L-4-fluorophenylalanine; Nal, L-2-naphthylalanine; nal, D-2-naphthylalanine; Nle, L-norleucine; Orn, L-ornithine; Phg, L-phenylglycine.

[2] Etf-1 binding affinity was measured by the fluorescence polarization (FP) assay, using peptides labeled with FAM through a C-terminal β-Ala-β-Ala-Lys linker sequence. ND, not determined.

Supplementary Table S2: Sequences and Etf-1-binding affinities of B7 analogs through alanine scan and optimization

Peptide ID	Sequences [1]	K_D (μM) [2]
**B7**	TMA-Tyr-His-Gln-pro-Ser-leu-Dap-Phe-nal-arg-Arg-arg-Arg-Dap	6.5 ± 0.7
**B7-1**	TMA-Tyr-His-Gln-pro-Ser-ala-Dap-Phe-nal-arg-Arg-arg-Arg-Dap	18.9 ± 6.9
**B7-2**	TMA-Tyr-His-Gln-pro-Ala-leu-Dap-Phe-nal-arg-Arg-arg-Arg-Dap	6.7 ± 0.8
**B7-3**	TMA-Tyr-His-Gln-ala-Ser-leu-Dap-Phe-nal-arg-Arg-arg-Arg-Dap	5.8 ± 0.5
**B7-4**	TMA-Tyr-His-Ala-pro-Ser-leu-Dap-Phe-nal-arg-Arg-arg-Arg-Dap	7.4 ± 0.9
**B7-5**	TMA-Tyr-Ala-Gln-pro-Ser-leu-Dap-Phe-nal-arg-Arg-arg-Arg-Dap	10.9 ± 1.5
**B7-6**	TMA-Ala-His-Gln-pro-Ser-leu-Dap-Phe-nal-arg-Arg-arg-Arg-Dap	11.9 ± 3.4
**B7-7**	TMA-Tyr-His-Gln-pro-Ser-leu-Dap-Ala-nal-arg-Arg-arg-Arg-Dap	NA
**B7-8**	TMA-Tyr-His-Gln-pro-Ser-leu-Dap-Phe-ala-arg-Arg-arg-Arg-Dap	NA
**B7-9**	TMA-Tyr-His-Gln-pro-Ser-leu-Dap-Phe-nal-ala-Arg-arg-Arg-Dap	30 ± 7
**B7-10**	TMA-Tyr-His-Gln-pro-Ser-leu-Dap-Phe-nal-arg-Ala-arg-Arg-Dap	30 ± 3
**B7-11**	TMA-Tyr-His-Gln-pro-Ser-leu-Dap-Phe-nal-arg-Arg-ala-Arg-Dap	28 ± 6
**B7-12**	TMA-Tyr-His-Gln-pro-Ser-leu-Dap-Phe-nal-arg-Arg-arg-Ala-Dap	15 ± 2
**B7-13**	TMA-Tyr-His-Gln-pro-Ser-leu-Dap-4-MePhe-nal-arg-Arg-arg-Arg-Dap	6.3 ± 0.8
**B7-14**	TMA-Tyr-His-Gln-pro-Ser-leu-Dap-Phg-nal-arg-Arg-arg-Arg-Dap	5.8 ± 0.9
**B7-15**	TMA-Tyr-His-Gln-pro-Ser-leu-Dap-HomoPhe-nal-arg-Arg-arg-Arg-Dap	3.4 ± 0.6
**B7-16**	TMA-Tyr-His-Gln-pro-Ser-leu-Dap-4-ClPhe-nal-arg-Arg-arg-Arg-Dap	2.0 ± 0.2
**B7-17**	TMA-Tyr-His-Gln-pro-Ser-leu-Dap-3-ClPhe-nal-arg-Arg-arg-Arg-Dap	3.4 ± 0.4
**B7-18**	TMA-Tyr-His-Gln-pro-Ser-leu-Dap-Fpa-nal-arg-Arg-arg-Arg-Dap	3.0 ± 0.5
**B7-19**	TMA-Tyr-His-Gln-pro-Ser-leu-Dap-Cl_2Phe-nal-arg-Arg-arg-Arg-Dap	1.6 ± 0.2
**B7-20**	TMA-Tyr-His-Gln-pro-Ser-leu-Dap-4-CF_3Phe-nal-arg-Arg-arg-Arg-Dap	1.8 ± 0.2
**B7-21**	TMA-Tyr-His-Gln-pro-Ser-leu-Dap-Nal-nal-arg-Arg-arg-Arg-Dap	1.9 ± 0.2
**B7-22**	TMA-Tyr-His-Gln-pro-Ser-leu-Dap-4-ClPhe-bta-arg-Arg-arg-Arg-Dap	5.7 ± 0.9
**B7-23**	TMA-Tyr-His-Gln-pro-Ser-leu-Dap-4-ClPhe-1-nal-arg-Arg-arg-Arg-Dap	4.7 ± 0.5

1 Abbreviations: Three-letter abbreviations with capitalized first letter are used for L-amino acids, while three-letter abbreviations in lowercase letters for D-amino acids. Dap, 2,3-Diaminopropionic acid; Nal, L-2-naphthylalanine; nal, D-2-naphthylalanine; Phg, L-phenylglycine; HomoPhe, L-homophenylalanine; 4-MePhe, L-4-methylphenylalanine; 3-ClPhe, L-3-chlorophenylalanine; 4-ClPhe, L-4-chlorophenylalanine; Fpa, L-4-fluorophenylalanine; Cl_2Phe, L-3,4-dichlorophenylalanine; 4-CF_3Phe, L-4-trifluoromethyl-phenylalanine; bta, 3-(3-benzothienyl)-D-alanine; 1-nal, D-1-naphthylalanine.

2 Etf-1 binding was measured by FP assay, using peptides labeled with FAM through a C-terminal β-Ala-β-Ala-Lys linker sequence. NA, no significant binding.

Supplementary Table S3. Sequences and Etf-1-binding affinities of macrocyclic peptides identified from the second-generation library screening

Peptide ID	Sequence [1]	K_D (µM) [2]
**B7-95-1**	TMA-Tyr-Asp-Orn-isoAsp-glu-Fpa-leu-Dap-4-ClPhe-nal-arg-Arg-arg-Arg-Dap	3.2
**B7-95-2**	TMA-Tyr-val-Asp-Asp-Orn-Fpa-leu-Dap-4-ClPhe-nal-arg-Arg-arg-Arg-Dap	0.74 ± 0.06
**B7-131-1**	TMA-Tyr-asn-nal-Ser-Phg-leu-leu-Dap-4-ClPhe-nal-arg-Arg-arg-Arg-Dap	1.7 ± 0.2
**B7-131-2**	TMA-Tyr-His-nal-Gln-Ser-Fpa-leu-Dap-4-ClPhe-nal-arg-Arg-arg-Arg-Dap	8.8 ± 0.9
**B7-131-3**	TMA-Tyr-Phg-Orn-ala-Ieu-Orn-leu-Dap-4-ClPhe-nal-arg-Arg-arg-Arg-Dap	ND
**B7-131-4**	TMA-Tyr-Fpa-Asp-Phg-tyr-isoAsp-leu-Dap-4-ClPhe-nal-arg-Arg-arg-Arg-Dap	~1.5
**B7-131-5**	TMA-Tyr-His-pro-nal-Asp-Ala-leu-Dap-4-ClPhe-nal-arg-Arg-arg-Arg-Dap	2.9 ± 0.4
**B7-131-6**	TMA-Tyr-Pip-glu-Asp-nal-Gly-leu-Dap-4-ClPhe-nal-arg-Arg-arg-Arg-Dap	ND
**B7-133-1**	TMA-Tyr-Arg-(cis-AcPc)-Gly-Asp-leu-Dap-4-ClPhe-nal-arg-Arg-arg-Arg-Dap	6.1 ± 0.9
**B7-133-2**	TMA-Tyr-ala-Asp-tyr-ala-leu-Dap-4-ClPhe-nal-arg-Arg-arg-Arg-Dap	8.2 ± 1.2
**B7-133-3**	TMA-Tyr-phe-Asp-Nle-asn-leu-Dap-4-ClPhe-nal-arg-Arg-arg-Arg-Dap	2.4 ± 0.3
**B7-133-4**	TMA-Tyr-Nle-glu-Gln-Gly-leu-Dap-4-ClPhe-nal-arg-Arg-arg-Arg-Dap	6.5 ± 0.1
**B7-133-5**	TMA-Tyr-isoAsp-His-Asp-His-leu-Dap-4-ClPhe-nal-arg-Arg-arg-Arg-Dap	>10
**B7-133-6**	TMA-Tyr-Phg-His-Orn-Arg-leu-Dap-4-ClPhe-nal-arg-Arg-arg-Arg-Dap	ND
**B7-133-7**	TMA-Tyr-Trp-Nle-Phg-Gly-leu-Dap-4-ClPhe-nal-arg-Arg-arg-Arg-Dap	NA
**B7-133-8**	TMA-Tyr-nal-Gln-homoAla-pro-leu-Dap-4-ClPhe-nal-arg-Arg-arg-Arg-Dap	1.3 ± 0.1
**B7-133-9**	TMA-Tyr-val-leu-tyr-Fpa-leu-Dap-4-ClPhe-nal-arg-Arg-arg-Arg-Dap	2.2 ± 0.3

[1] In addition to those listed in the footnote of Tables 1 and 2, the abbreviations for nonproteinogenic amino acids are: homoAla, D-β-homo-alanine; Pip, L-homo-proline; cis-AcPc, cis-2-aminocyclopentyl-carboxylic acid; isoAsp, aspartic acid α-tert-butyl ester.

[2] Etf-1 binding was measured by FP assay, using peptides labeled with FAM through a C-terminal β-Ala-β-Ala-Lys linker sequence. ND, not determined; NA, no significant binding.

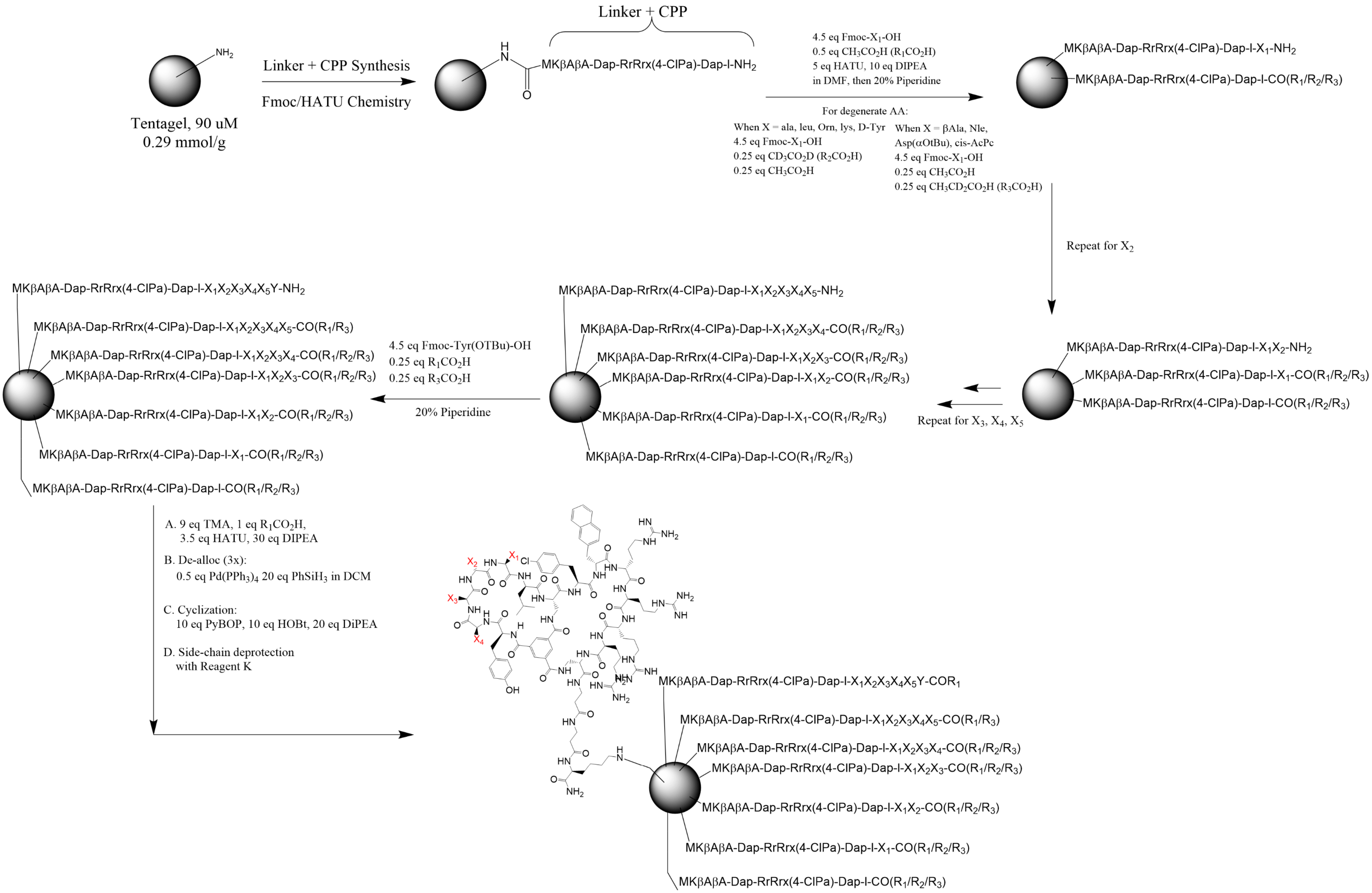

Figure S1. Synthesis scheme for the second-generation macrocyclic peptide library.

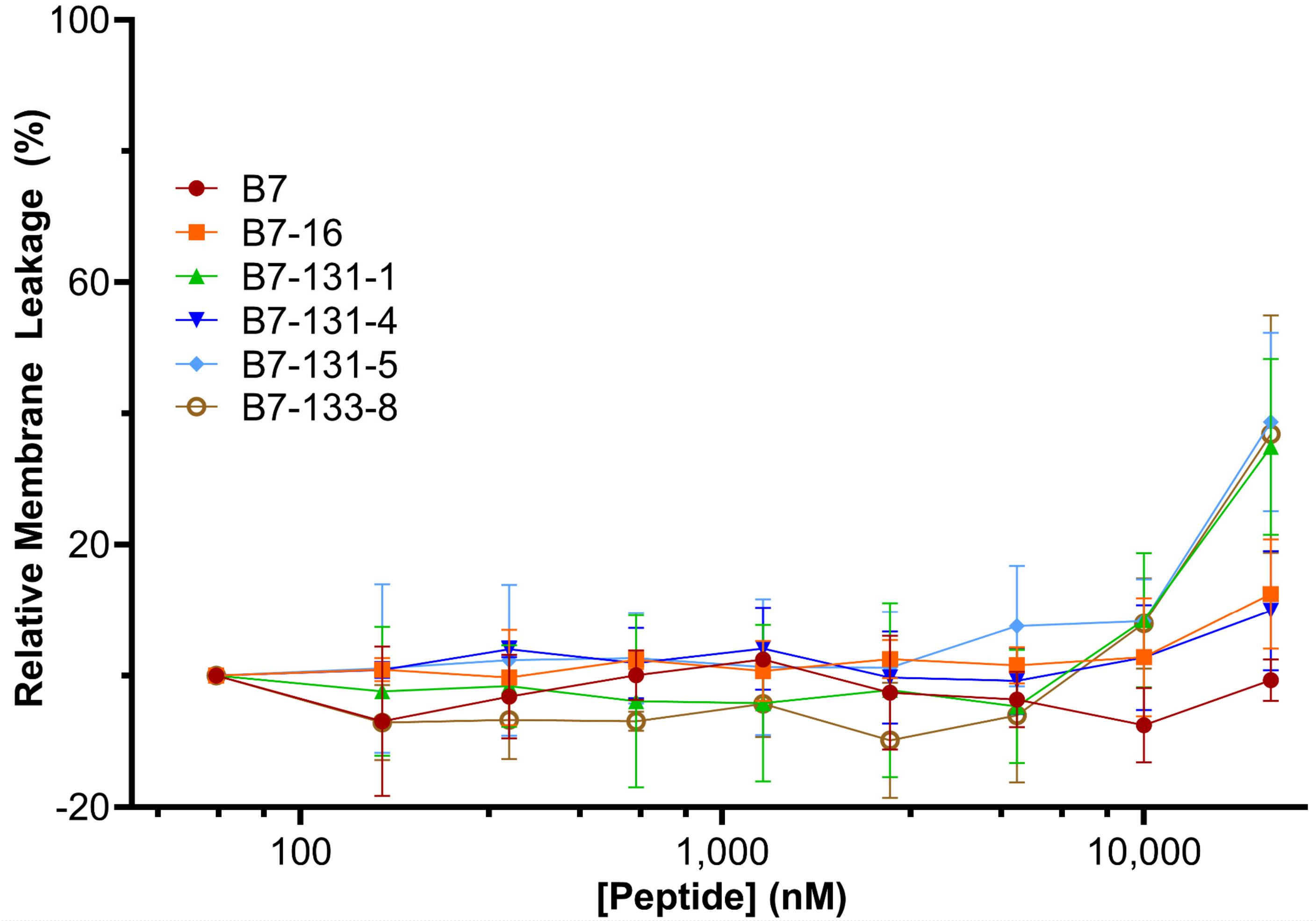

Figure S2. Determination of plasma membrane damage by macrocyclic peptides using LDH release assay.
THP-1 cells were harvested and seeded into clear 96-well plates at a final density of 5 × 10^3 cells/well in complete growth media and incubated overnight at 37 °C and 5% CO_2. Peptides were serially diluted in DPBS and added to each well with a constant final concentration of 0.5% DMSO (v/v). Control wells contained 10 μL of lysis buffer, cell-free complete growth media, or positive LDH control. Following incubation at 37 °C and 5% CO_2 for 45 min to 75 min, cells were spun down and 50 μL of growth medium was withdrawn from each well, transferred to a clear 96-well plate, mixed with 50 μL of LDH substrate mix, and incubated at RT for 30 min with gentle mixing. Finally, 50 μL of 1 N HCl was added to each well, and the absorbances at 490 and 680 nm were immediately measured on a Tecan Infinite M1000 plate reader. Percent relative membrane leakage was calculated using the following equation [($Abs_{compound}$ – $Abs_{untreated}$) / ($Abs_{lysed\ cells}$ – $Abs_{untreated}$) × 100] and plotted using GraphPad Prism.

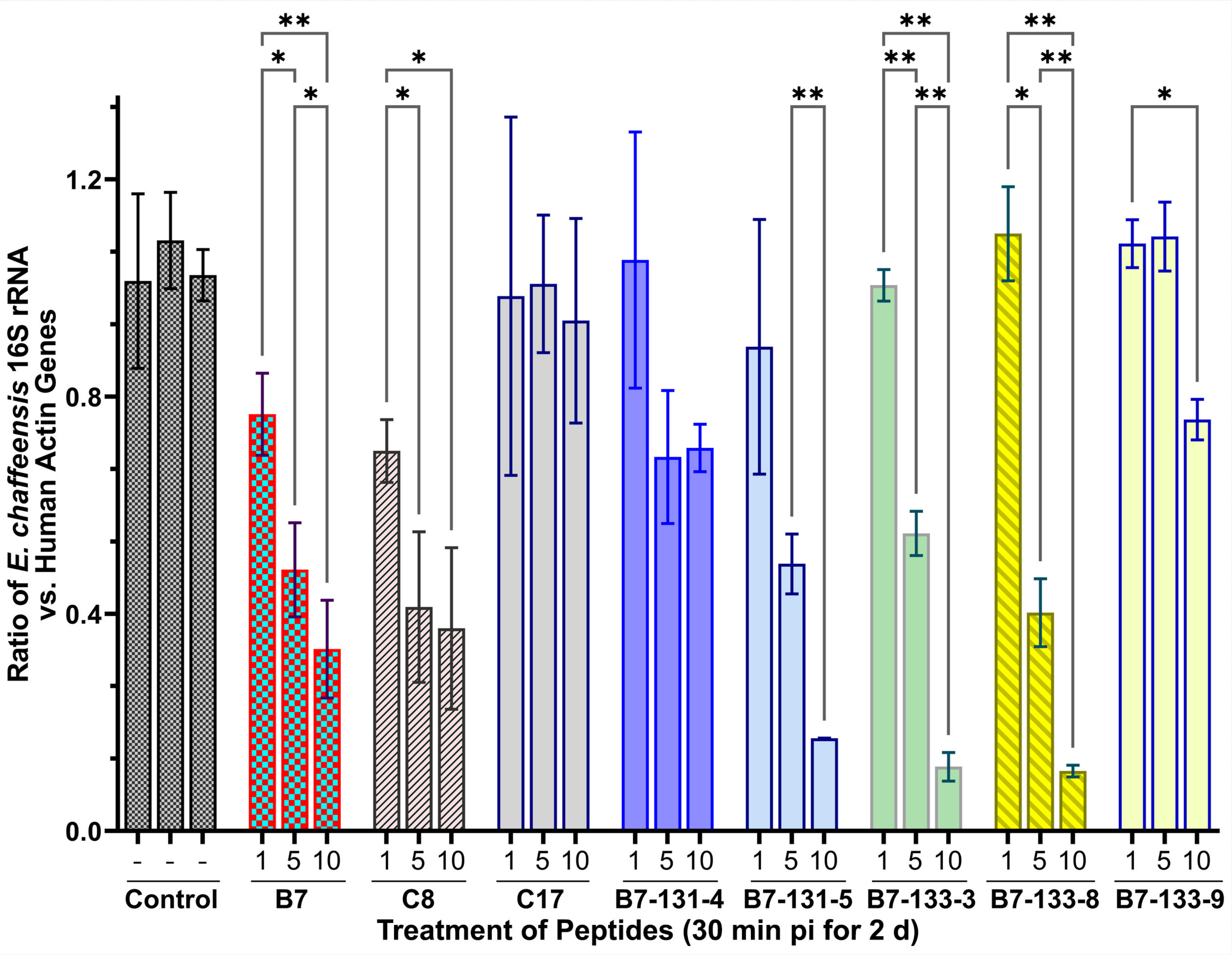

Figure S3. Effects of macrocyclic peptides on *E. chaffeensis* infection of THP-1 cells.
E. chaffeensis-infected THP-1 cells at 3 h pi were incubated with 1, 5, or 10 μM Etf-1 bicyclic peptides, or DMSO control for 2d. DNA was extracted from each sample using Qiagen blood mini kit, and subjected to qPCR analysis using *E. chaffeensis* 16S rRNA gene normalized to human *ACTIN* gene. **, $P < 0.01$; *, $P < 0.05$; one-way ANOVA.

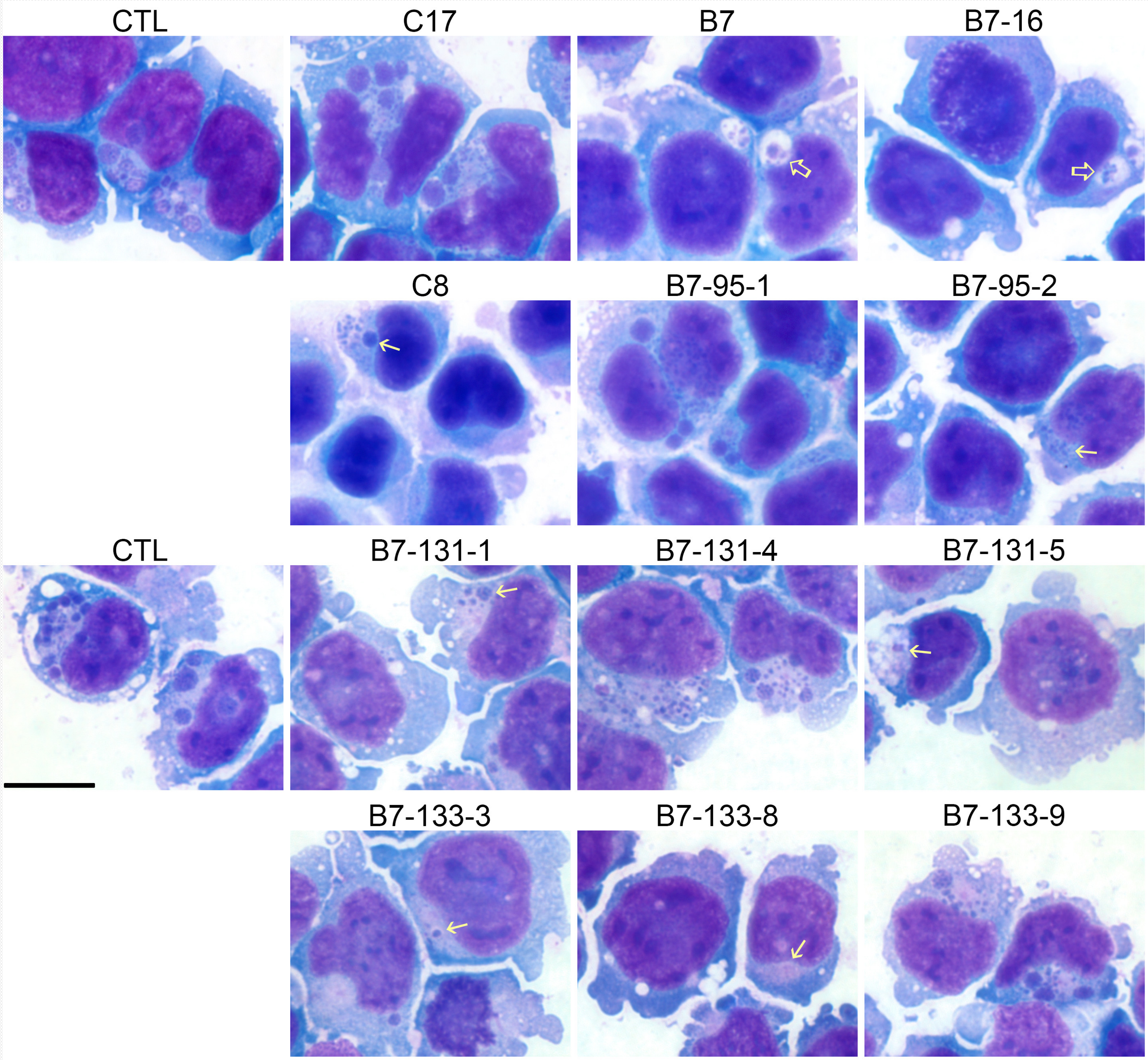

Figure S4. Effects of Etf-1 binding peptides on *E. chaffeensis* Infection. *E. chaffeensis*-infected THP-1 cells were incubated with 10 μM Etf-1-binding peptides or DMSO control (CTL) at 3 h pi for 2 d. Cells were cytospun onto slides and stained by Diff-Quik. Representative images were shown for each treatment sample from at least three independent experiments with similar results. In control groups or peptides that did not inhibit *E. chaffeensis* infection (C17, B7-95-1, and B7-131-4), 2 – 5 large spherical membrane-bound vacuoles called morulae in the cytoplasm can be identified per cell, each contained more than 10 *E. chaffeensis* organisms stained dark blue to purple. Peptides B7 and B7-16 significantly inhibited infection, resulting in an enlarged intra-cytoplasmic vacuole containing few dark and shrunken *E. chaffeensis* organisms (open arrows). Other peptides that inhibited *E. chaffeensis* infection, including C8, B7-95-2, B7-131-1, 3-151-8, 1-133-3, and B7-133-8, had less and smaller morulae containing fewer numbers of *E. chaffeensis* organisms (thin arrows). Bar, 10 μm.

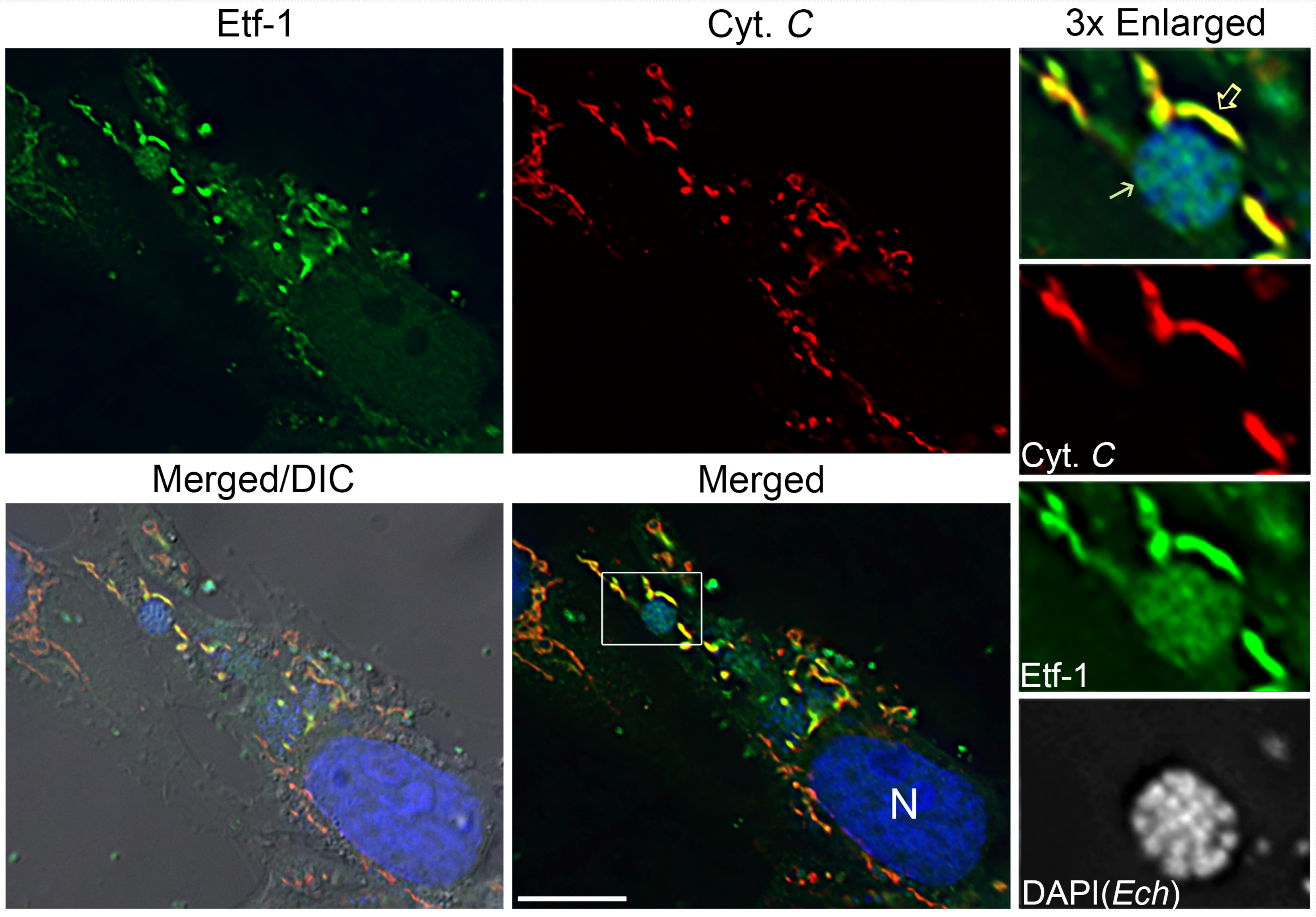

Figure S5. Etf-1 localization at host mitochondria and *E. chaffeensis*-containing inclusion membranes. RF/6A cells were seeded onto coverslips in a 12-well plate and infected with *E. chaffeensis* for 2 d. Cells were fixed, labeled with mouse anti-cytochrome *C* (Cyt. *C*) and rabbit anti-Etf-1 in PGS for 1 h, then with AF488-conjugated goat anti-rabbit and AF555-goat anti-mouse IgG in PGS for 1 h. DAPI was used to label host nuclei and *E. chaffeensis* DNA (pseudocolored grey in 3x enlarged images). DeltaVision microscope. *E. chaffeensis* organisms were shown as round to pleomorphic cocci that further clustered as spherical morulae in 3× enlarged images (blue or grey-colored in merged or DAPI channels, respectively). Open arrow, filamentous-shaped mitochondria as indicated by cytochrome *C* labeling and colocalized with native Etf-1 protein; thin arrow, *E. chaffeensis*-containing morulae. Merged, merge of fluorescence images; DIC, differential interference contrast image; Bar, 10 µm. Images are representative from at least 3 independent experiments.

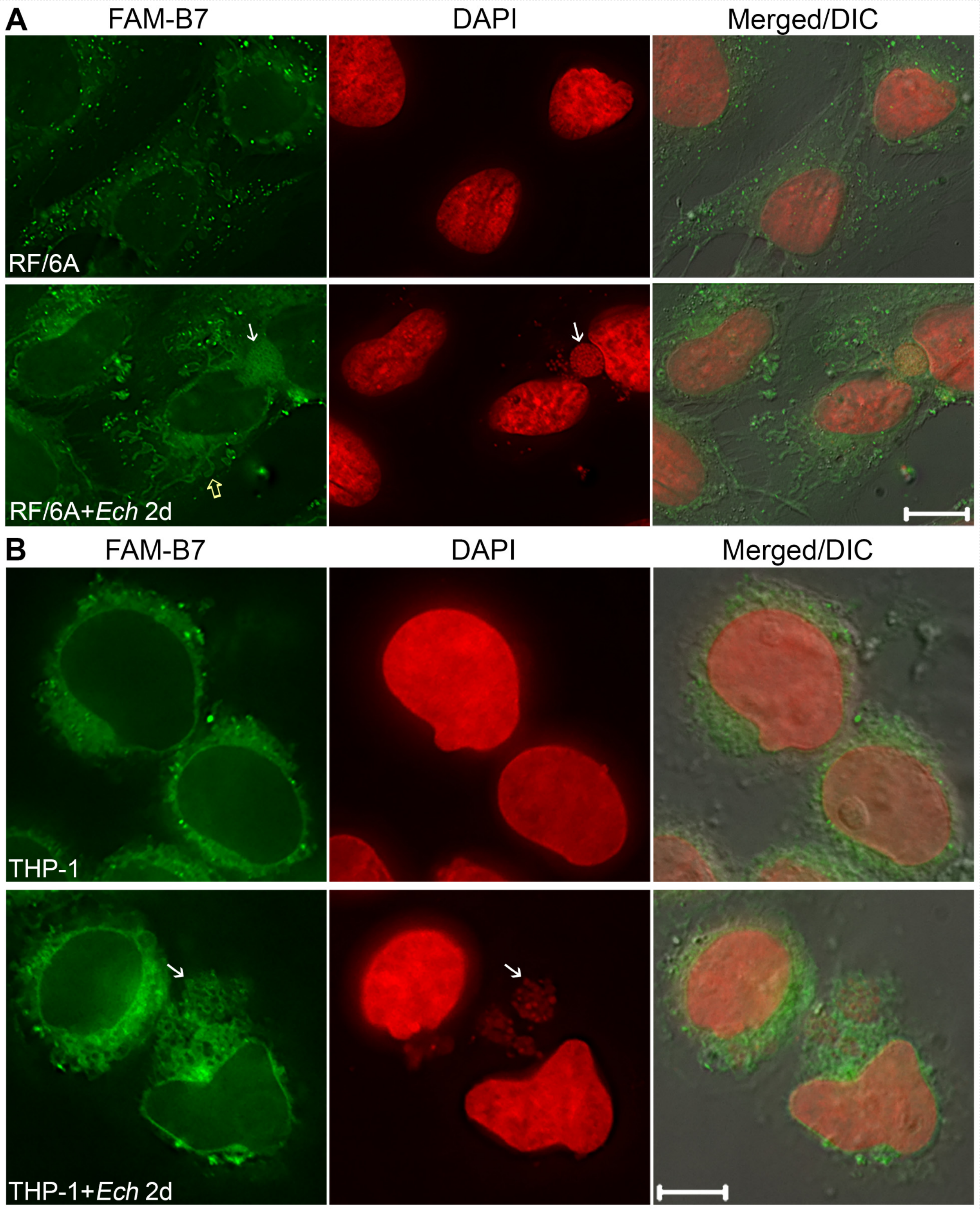

Figure S6. Localization of FAM-B7 in uninfected or *E. chaffeensis*-infected cells.
Uninfected or *E. chaffeensis (Ech)*-infected RF/6A (**A**) or THP-1 cells (**B**) at 2 d pi were incubated with 2 μM FAM-B7 peptide for 2 h. Cells were fixed in 4% PFA, stained with DAPI (pseudocolored in red), and observed under DeltaVision microscope. *E. chaffeensis* organisms were shown as round to pleomorphic cocci that further clustered as spherical morulae (red-colored in DAPI channels). Open arrow (A), filamentous-shaped mitochondria in RF/6A cells; thin arrows (A and B), *E. chaffeensis*-containing morulae. Merged/DIC, merged fluorescence and differential interference contrast images; Bar, 10 μm. Images are representative from at least 3 independent experiments.

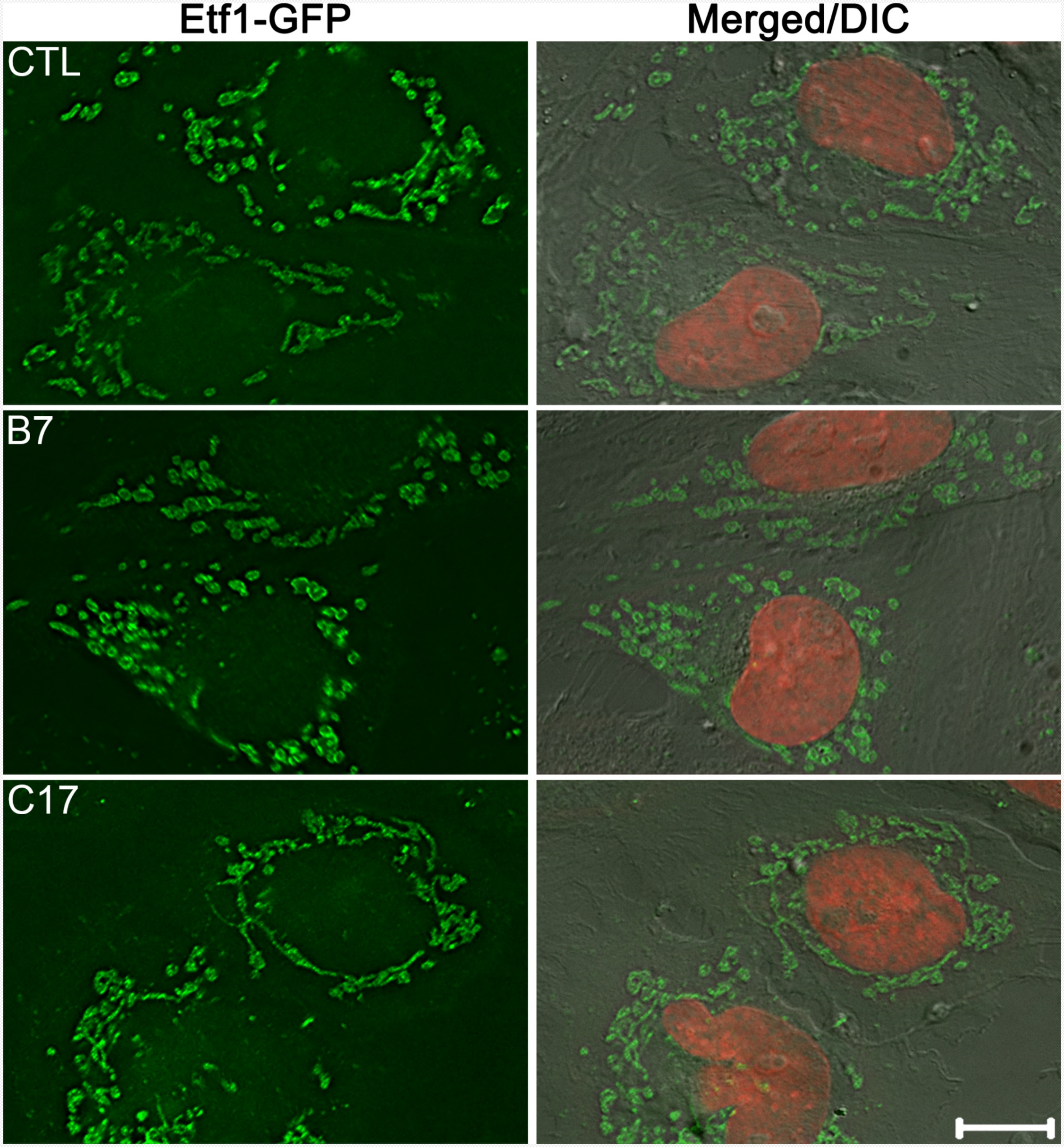

Figure S7. Localizations of Etf1-GFP at mitochondria in transfected RF/6A cells were not affected by treatments of macrocyclic peptides.
RF/6A cells were transfected with Etf1-GFP plasmids by electroporation, and seeded into a 6-well plate. At 16 h pt, cells were incubated with 10 μM of macrocyclic peptides (B7 or C17) for 2 d. Cells were fixed in 4% PFA and host nuclei were stained with 1 μg/ml Hoechst 33342 (pseudocolored red) for 10 min in PBS. DeltaVision microscope. Ectopically expressed Etf-1-GFP was localized to filamentous-shaped mitochondria (green), which was not affected by peptide treatment. Merged/DIC, merge of fluorescence and DIC images. Bar, 10 μm.
